# Hydrogen-Stabilized ScYNdGd Medium-Entropy Alloy for Hydrogen Storage

**DOI:** 10.1021/jacs.3c11943

**Published:** 2024-02-14

**Authors:** Mateusz Balcerzak, Jéssica Bruna Ponsoni, Hilke Petersen, César Menéndez, Jan Ternieden, Linda Zhang, Frederik Winkelmann, Kondo-Francois Aguey-Zinsou, Michael Hirscher, Michael Felderhoff

**Affiliations:** † Heterogeneous Catalysis Department, Max-Planck-Institut für Kohlenforschung, Mülheim an der Ruhr 45470, Germany; ‡ Institute of Materials Science and Engineering, Poznan University of Technology, Poznan 61-138, Poland; § Graduate Program in Materials Science and Engineering (PPGCEM/UFSCar), Federal University of Sao Carlos, São Carlos, São Paulo CEP 13565-905, Brazil; ∥ MERLin, School of Chemistry, University of Sydney, Sydney, NSW 2006, Australia; ⊥ Max-Planck-Institut für Intelligente Systeme, Stuttgart 70569, Germany; # Advanced Institute for Materials Research, Tohoku University, Katahira 2-1-1, Aoba-ku, Sendai 980-8577, Japan

## Abstract

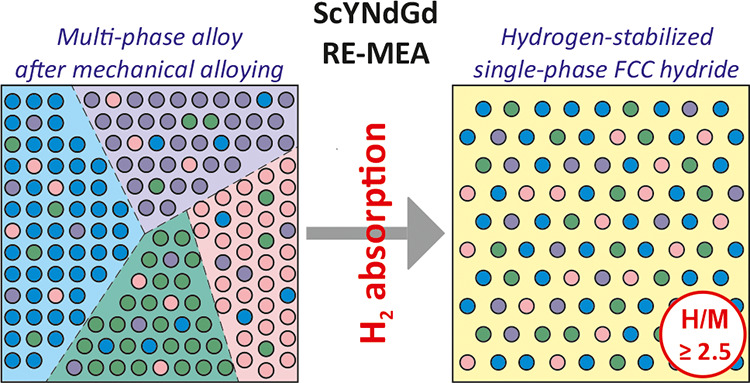

The research on the functional properties of medium- and high-entropy alloys (MEAs and HEAs) has been in the spotlight recently. Many significant discoveries have been made lately in hydrogen-based economy-related research where these alloys may be utilized in all of its key sectors: water electrolysis, hydrogen storage, and fuel cell applications. Despite the rapid development of MEAs and HEAs with the ability to reversibly absorb hydrogen, the research is limited to transition-metal-based alloys that crystallize in body-centered cubic solid solution or Laves phase structures. To date, no study has been devoted to the hydrogenation of rare-earth-element (REE)-based MEAs or HEAs, as well as to the alloys crystallizing in face-centered-cubic (FCC) or hexagonal-close-packed structures. Here, we elucidate the formation and hydrogen storage properties of REE-based ScYNdGd MEA. More specifically, we present the astounding stabilization of the single-phase FCC structure induced by the hydrogen absorption process. Moreover, the measured unprecedented high storage capacity of 2.5 H/M has been observed after hydrogenation conducted under mild conditions that proceeded without any phase transformation in the material. The studied MEA can be facilely activated, even after a long passivation time. The results of complementary measurements showed that the hydrogen desorption process proceeds in two steps. In the first, hydrogen is released from octahedral interstitial sites at relatively low temperatures. In the second, high-temperature process, it is associated with the desorption of hydrogen atoms stored in tetrahedral sites. The presented results may impact future research of a novel group of REE-based MEAs and HEAs with adaptable hydrogen storage properties and a broad scope of possible applications.

## Introduction

The efficient operation of a hydrogen-based economy requires a high degree of technological maturity in its three key areas: cheap production of green hydrogen through water electrolysis, effective and safe hydrogen storage, and efficient hydrogen end-use in electricity generation or industrial applications. These areas are vigorously developing through new engineering solutions, but rapid technological progress is limited by the availability of novel materials that would far outperform the currently used ones. Medium-entropy alloys (MEAs) and high-entropy alloys (HEAs) can remedy this problem.

MEAs and HEAs are, by arbitrary definition, single-phase solid solution alloys composed of elements with a molar fraction of 5–35 atomic %.^1^ The difference between these two similar groups of alloys lies in the configurational entropy value (Δ*S*_config_), which is at least partly responsible for stabilizing a single-phase structure. For MEAs and HEAs, Δ*S*_config_ is in the ranges of 0.69R < Δ*S*_config_ < 1.61*R* and ≥1.61*R*, respectively (where *R* is a gas constant).^2,3^ This essentially means that in equimolar alloys, MEAs are composed of three to four elements, while HEAs are of at least five. In recent years, there has been a noticeable interest in using entropy-stabilized alloys as electrochemical catalysts fixed on both anode and cathode sites in either water electrolyzers or fuel cells working in both alkaline or acidic conditions.^2,4−6^ It has been demonstrated that their exceptional catalytic properties are coupled with the fine-tailoring of binding energies (due to the “cocktail” effect) that can synergistically modify short- and long-range interactions of the active centers with neighboring atoms. Moreover, the chemical stability of these catalysts is improved by the sluggish diffusion effect resulting from the severe lattice distortion of these alloys.^7,8^

The first research on the hydrogen storage properties of TiVZrNbHf, published in 2016, was an impulse for dynamic development of MEAs and HEAs for hydrogen storage applications.^9^ Since then, more and more scientific output has been released every year.^10^ To date, the research in this field has focused on exploring the effect of lattice strain,^11,12^ valence electron concentration (VEC),^13,14^ degree of occupation of the interstitial sites,^15,16^ chemical composition,^17,18^ an increase of gravimetric storage capacity by a decrease of alloy‘s molar weight,^19−21^ machine/statistical learning models,^22^ or thermodynamic design of alloys according to their structural stability and hydrogen storage properties.^23−25^ Furthermore, the research on chemically complex alloys has been further expanded to single-phase intermetallic and amorphous systems, which, by definition, due to the lack of a solid solution phase, cannot be called entropy-stabilized ones.^26−29^ It seems that one of the greatest promises of these materials will be the designability of chemical composition and crystal structure according to the hydrogen storage requirements (e.g., storage capacity, absorption/desorption hydrogen pressure) with particular emphasis on the alloy’s price reduction.

Nevertheless, the current state of the knowledge on MEA’s and HEA’s hydrogen storage properties is limited to the systems that crystallize in body-centered cubic (BCC) solid solution. There is no published research on entropy-stabilized alloys (in the metallic state) with face-centered cubic (FCC) or hexagonal-close-packed (HCP) solid solution structures that were evaluated in this regard. Moreover, the research has focused on alloys composed of mainly transition metals (TM), and the influence of rare-earth elements (REE), such as Y, Sc, or La, on the hydrogenation and dehydrogenation properties of MEAs and HEAs has not been addressed in the literature so far. It is worth mentioning that, in general, REE-MEAs and HEAs are strongly underrepresented compared to other groups of entropy-stabilized alloys (the limited research to date has focused mostly on their mechanical and magnetic properties).^30,31^ However, REE should be considered attractive elements for hydrogen storage materials, as most of them are capable of prompt hydrogen absorption at room temperature under pressures well below 1 atm. REE-based hydrides also demonstrate intriguing structural phase transitions upon hydrogenation. The metallic REEs with mostly HCP structure undergo the phase transformation toward FCC structure (crystallizing in cubic CaF_2_-type structure) when the hydrogen concentration reaches MH_2_ (2 H/M), where M stands for the metal atom. The further increase of H concentration to approximately the concentration of MH_3_ trihydride (3 H/M) results in either restabilization of the original HCP structure or maintenance of the FCC dihydride structure, depending on the REE.^32,33^

Herein, we enter the vast, undeveloped area of REE-based MEAs and HEAs, opening a new field of research devoted primarily (but not exclusively) to their hydrogen storage properties. The synthesis of the ScYNdGd alloy was approached by employing a facile mechanical alloying (MA) method. The set of complementary experiments showed the formation of single-phase FCC MEA hydride that has been firmly stabilized by hydrogen atoms. Furthermore, the unique properties of the novel alloy and its hydride were presented and discussed based on both experimental and computational methods.

## Results and Discussion

### Mechanical Alloying and Thermal Stability

The ScYNdGd MEA was synthesized using pure ScH_2_, YH_2_, Nd, and GdH_2_ (Figure S1, Table S1). The use of brittle hydrides facilitates MA, preventing severe cold welding (a process of joining metal surfaces together that requires little or no heat) and the accumulation of processed material on the milling media. The starting powders mixed in equimolar composition (considering metal atoms) were processed in a planetary mill under argon. The milling was interrupted each hour to dissipate the generated heat and monitor the MA progress by XRD. The final MA time was 5 h.

**Figure 1:**
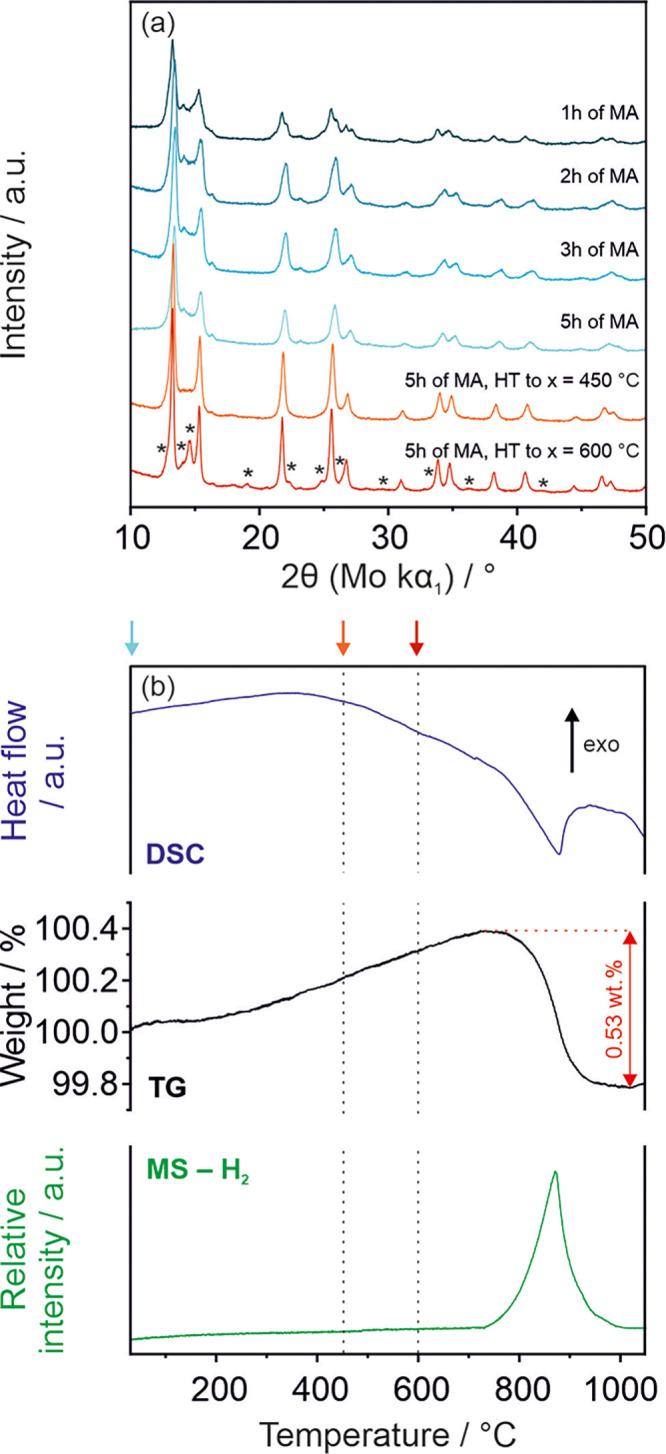
(a) XRD patterns obtained for Sc–Y–Nd–Gd element mixture at different stages of the MA process and of the sample after 5 h MA heat-treated (HT) via DSC/TG experiments (30-*x* °C, 5 °C/min) that ended at different temperatures (*x*); * indicates the positions of reflections of the recrystallized HCP phase. (b) DSC, TG, and MS results obtained for as-synthesized (after 5h MA) ScYNdGd alloy (5 °C/min). The arrows point to the maximum temperatures of HT after which the structure was studied by XRD.

The XRD patterns and their fittings (Pawley fits) are presented in Figures 1a and S2, respectively. The collected data revealed that, independently of the total milling time, the material crystallizes as a multiphase alloy composed of at least four cubic phases (Figure S2). Moreover, a tendency toward forming single-phase solid solution MEAs was not observed. Considering that the semiempirical parameters strongly indicate the formation of MEA for ScYGdNd composition (see Chapter 3 in the Supporting Information), the formation of a multiphase instead of a single-phase structure must be connected to using hydrides as the starting materials. The high stability of hydrides (see the thermal stability in Figure S1 and highly negative enthalpies of formation of the binary metal hydrides)^34^ to a great extent prevents materials from complete reaction with each other.

The SEM imaging of the as-synthesized alloy revealed the formation of irregular ScYNdGd particles of different shapes and sizes varying from a few to 40 μm (Figure S3). Moreover, further SEM analysis showed homogeneous alloy density in both the surface and bulk areas (Figure S4). The detailed STEM analysis showed a defined poly nanocrystalline structure in the bulk and highly deformed, amorphous surface layer, which is typical for the highly energetic MA of metals (Figure S5). The EDX analysis performed at low magnifications revealed an even elemental distribution in the alloy particles (Figure S6). However, the more detailed EDX analysis of separated alloy particles showed that for some of them a locally changed chemical composition can also be observed (Figure S7). These inhomogeneities correspond well with the described multiphase structure of the synthesized alloy, where each phase must differ from the other in its chemical composition.

The chemical composition of the as-synthesized alloy calculated based on the EDX data is Sc_0.23_Y_0.22_Nd_0.28_Gd_0.27_ and is close to the desired one. The slight discrepancy between the designed and actual chemical compositions of the alloy is discussed in detail in Chapter 4 in the Supporting Information. For the sake of simplicity, in the following part of the paper, we name the as-synthesized alloy ScYNdGd (even if we know that this is not a genuinely equimolar alloy).

The thermal stability test of the as-synthesized alloy, done by using TG/DSC/MS setup (Figure 1b), showed the presence of hydrogen (0.53 wt %) that desorbed from an alloy in an endothermic reaction at 700–1000 °C. The hydrogen atoms originate from the starting materials used for the MA. The ex situ XRD patterns obtained after TG/DSC/MS experiments, which ended at various temperatures, showed that the structure of the as-synthesized alloy is stable at least up to 450 °C (no significant changes have been observed). However, further heating to 600 °C causes segregation/recrystallization of the HCP structure with a lattice parameter close to the metallic yttrium.^35^

To summarize, the as-synthesized, partly hydrogenated ScYNdGd alloy crystallized as a multiphase and did not form MEA. The alloy particles were covered by an amorphous surface layer. Despite the overall uniform distribution of elements, local inhomogeneities of the chemical composition can be found. The alloy’s crystal structure is stable at 450 °C.

### First Hydrogenation/Dehydrogenation Experiments

**Figure 2:**
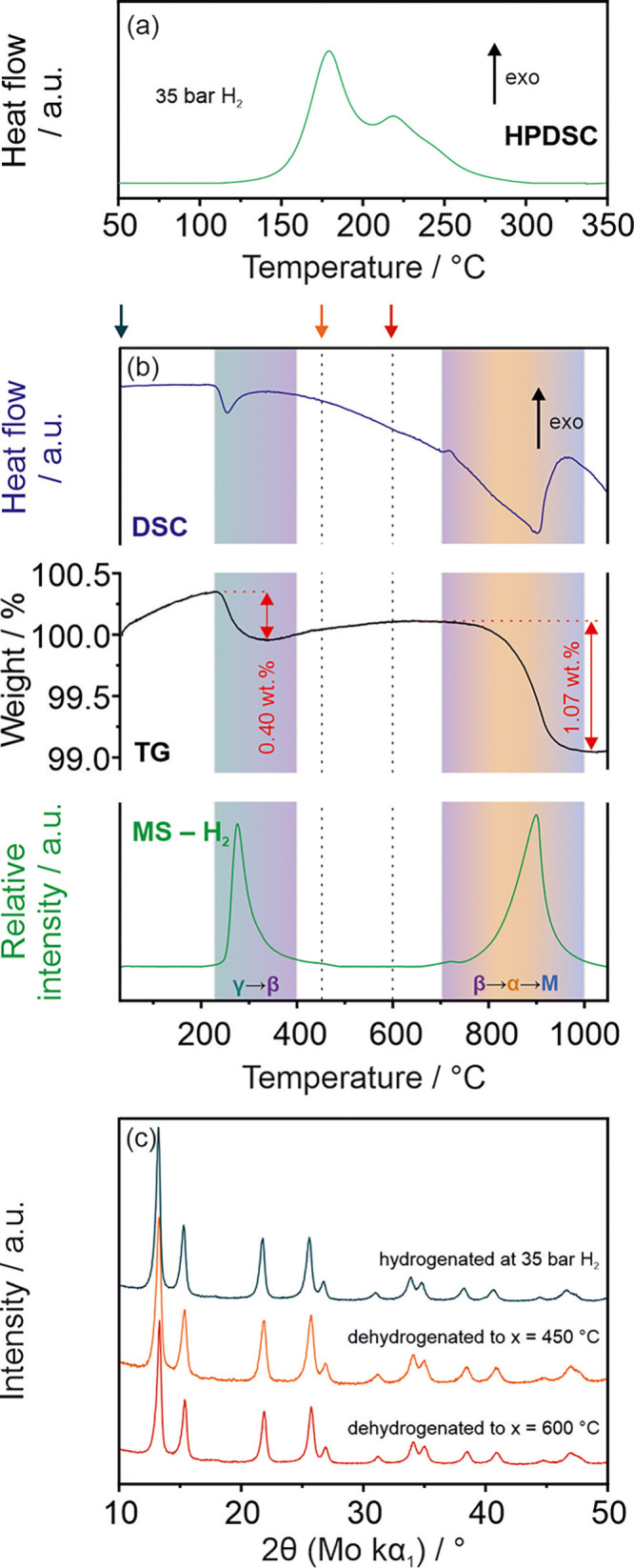
(a) Results of HPDSC hydrogenation experiments performed at 35 bar H_2_ (30–350 °C, 10 °C/min) (b) Results of DSC, TG, and MS (H_2_) dehydrogenation experiments performed on the sample hydrogenated at 35 bar H_2_ using HPDSC. The arrows point to the maximum temperatures of HT, after which the structure has been studied by XRD. (c) XRD patterns obtained for ScYNdGd alloy after hydrogenation at 35 bar H_2_ (using HPDSC) and after its DSC/TG/MS dehydrogenation experiments (30-*x* °C, 5 °C/min).

The as-synthesized alloy was used in an HPDSC experiment performed at 35 bar of H_2_ during heating from 30 to 350 °C. The results showed that the material absorbs hydrogen between 130 and 300 °C in a multistep reaction (Figure 2a). It is worth noting that the alloy does not require any pretreatment, such as degassing under vacuum, prior to the hydrogenation process. Detailed HPDSC experiments in which various H_2_ pressures were used (5–35 bar H_2_), showed that in all cases the hydrogenation process is comparable in terms of on-set temperature and curve profile (Figures S8 and S9, Chapter 5 in the Supporting Information).

The sample hydrogenated at 35 bar H_2_ (in the HPDSC experiment) was further used in the dehydrogenation experiments employing TG/DSC/MS setup (Figure 2b). The curve showed a decomposition process composed of a relatively low-temperature decomposition event at 220–400 °C and a high-temperature decomposition event at 700–1000 °C (similar to the as-synthesized alloy). The simple peak shapes, suggesting one decomposition process assigned to each endothermic event, are comparable to those observed for Y or Gd hydrides.^33^ According to the binary REE-H systems, the observed decomposition process should be assigned to the decomposition of trihydride (γ) to dihydride (β) in the first event and from β to hydride solution (α) and finally a metallic state of the alloy (M) in the second event.^33^ After hydrogenation at 35 bar H_2_, the amount of hydrogen stored in the β phase doubled compared to the as-synthesized alloy (0.53 wt %), reaching 1.07 wt %. Moreover, the additional amount of hydrogen (0.40 wt %) was stored in the γ phase, which was not observed in the as-synthesized alloy. The overall hydrogen storage capacity reached 1.47 wt %. Further experiments showed that the hydrogen storage capacity reaches similar values regardless of the hydrogenation pressure used (Figure S10 and Table S2).

Furthermore, the sample hydrogenated at 35 bar of H_2_ (in the HPDSC experiment) was studied by XRD to determine the structural changes caused by hydrogenation. Truly surprising is that the newly introduced amount of hydrogen stabilizes the MEA-based hydride structure that crystallizes in a single-phase FCC solid solution structure (Figures 2c and S11). No remnants of the multiphase structure of the as-synthesized alloy were observed. To the best of our knowledge, this is the first time this phenomenon has been observed for any entropy-stabilized alloys. Moreover, the obtained single-phase ScYNdGd alloy is the first REE-MEA that has been studied for hydrogen storage applications. According to the reported changes of cohesive energies upon hydrogenation of REE-elements, the origin for the stabilization of MEA should be connected to the formation of a large number of strong covalent bonds between the hydrogen and metal atoms.^36^

Moreover, the thermal stability tests (done ex situ using TG/DSC/MS setup) showed that the FCC structure is stabilized to at least 600 °C; the XRD results did not show any phase segregation as in the case of the as-synthesized alloy (Figures 1a and 2c). The shift of the reflection positions toward higher 2θ is visible for the XRD patterns obtained after HT. It is related to the cell volume’s shrinking resulting from partial hydrogen desorption (γ → β) (Figure 2c). It means that hydrogen is necessary to initiate REE-MEA stabilization but is not essential to maintain it.

As hydrogen tends to stabilize the single-phase MEA structure, we decided to test whether direct ball-milling synthesis under hydrogen pressure can lead to the formation of the same MEA structure. For this reason, we performed reactive mechanical alloying (RMA) under 30 bar H_2_ using the same milling parameters as in the MA. Although the XRD patterns show an evolution of the crystal structure at the beginning of the milling process (15–30 min), the changes in the structure between 30 and 60 min of RMA do not indicate the trend toward the formation of single-phase MEA (Figure S12a). The particularly probable reason for the lack of MEA formation is that as soon as the starting materials (as M or β phase) are in contact with hydrogen at the beginning of the RMA, the highly stable binary γ hydrides are formed (characterized by highly negative enthalpies of the binary metal hydrides formation), which are not prone to further interact with each other. The formation of the binary γ hydrides is confirmed by the two-step decomposition process visible on the TG curves obtained for the RMA material (Figure S12b). Interestingly, these RMA samples start to desorb hydrogen (within the first decomposition step) already around 50 °C within a long endothermic process hardly visible on the DSC curves. The analysis of the results discussed above shows that milling under Ar is a better strategy for obtaining REE-MEAs.

### Oxidation, Activation, and Reactivation of Alloy

**Figure 3:**
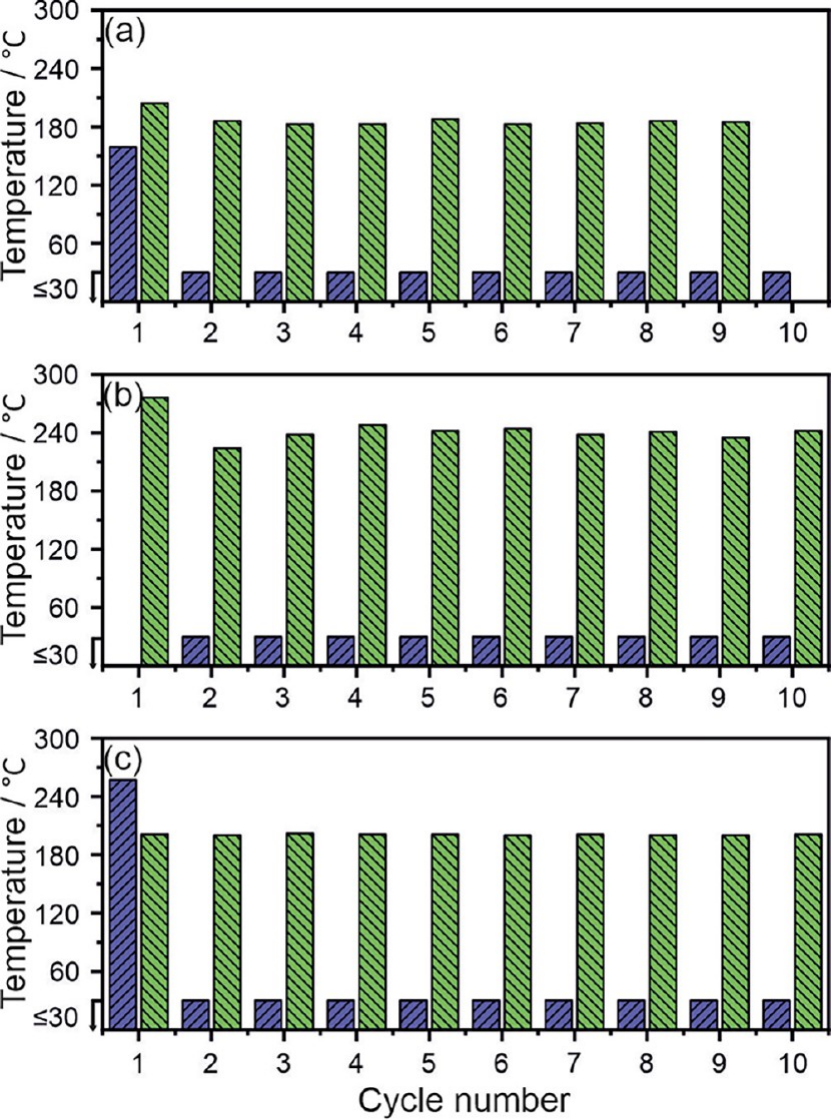
Hydrogenation (blue) and dehydrogenation (green) on-set temperatures in a function of hydrogenation/dehydrogenation cycles performed on ScYNdGd alloy: (a) after 5 h of MA, (b) in the activated and hydrogenated state left for 6-month-long surface oxidation in air, (c) in the activated and partly dehydrogenated state left for 6-month-long surface oxidation in air. The hydrogenation and dehydrogenation experiments were performed using HPDSC under 15 bar of H_2_ and Ar flow, respectively.

The surface of the as-synthesized alloy can be easily and extensively oxidized (Figures S13 and S14, Chapter 6 in the Supporting Information). Since most of the samples prone to surface oxidation that are considered for hydrogen storage-related applications require activation prior to achieving optimal hydrogenation properties, we conducted a series of experiments using HPDSC. The hydrogenation was done under 15 bar H_2_ (50–350 °C, 10 °C/min) and dehydrogenation under Ar flow (50–450 °C, 10 °C/min). As both experiments were performed one after another in the same apparatus (without handling of the sample and under hydrogen or argon atmosphere), the possibility of oxidation during and between the measurements was excluded. The only possible oxidation is related to the initial mounting of the sample in the apparatus. Figure 3a shows the on-set temperatures of both hydrogenation and dehydrogenation determined at different hydrogenation/dehydrogenation cycles performed on an as-synthesized sample. Since the maximum temperature of the HPDSC used is too low to desorb all of the hydrogen from the MEA, the cyclic hydrogenation/dehydrogenation studies focus on the on-set temperatures of the β ↔ γ reactions.

During the first hydrogenation and dehydrogenation processes, the on-set temperatures equaled 160 and 205 °C, respectively. Interestingly, the alloy could be fully hydrogenated in the second cycle already at the temperature of hydrogen insertion to the system (≤30 °C). The significant reduction of the hydrogenation on-set temperature is related to the alloy activation (Figure S15a,b, Chapter 6 in the Supporting Information). The later hydrogenation/dehydrogenation cycles (*n* < 2) show that the material absorbed hydrogen below 30 °C and that the desorption on-set temperature is around 185 °C. These results point out that the studied alloy can be easily activated within an uncomplicated hydrogenation/dehydrogenation process. As a result, the material is capable of absorbing hydrogen at room temperature. Considering the highly negative enthalpies of the formation of the binary metal hydrides of elements employed in this study, we could expect hydrogenation even below room temperature. It is worth mentioning that the XRD study performed on the sample after cyclic hydrogenation/dehydrogenation tests (which ended in the fully hydrogenated state) proved the stability of the FCC structure (Figure S16).

To check the influence of surface oxidation on hydrogenation/dehydrogenation reactions, two activated ScYNdGd MEA samples, one of which was fully hydrogenated and one partly dehydrogenated (after desorption up to 450 °C) were exposed to air for six months. Despite the alloy’s tendency to undergo strong surface oxidation (Figures S13 and S14), both samples showed excellent reactivation properties. In the case of the first, fully hydrogenated sample, the material was first dehydrogenated with the on-set temperature of 275 °C (Figure 3b). After this process, the sample did not require further activation and actively absorbed hydrogen at *T* ≤ 30 °C. The long-term surface oxidation affected solely the dehydrogenation on-set temperature, which after reactivation reached approximately 235 °C and was slightly higher compared to one obtained for the activated as-synthesized alloy (205 °C). Regarding the second, partly dehydrogenated sample, an elevated temperature of 255 °C was required to initiate the first hydrogenation reaction (Figure 3c). However, after only one cycle of hydrogenation and dehydrogenation, this sample could also absorb hydrogen at *T* ≤ 30 °C. The average dehydrogenation on-set temperature, which is very stable over the cycling, did not rise by more than 15 °C compared to the cycled as-synthesized sample. The above-discussed results clearly show that the studied ScYNdGd MEA can be easily activated and reactivated to absorb hydrogen at *T* ≤ 30 °C, regardless of the storage procedure.

### Detailed Hydrogenation/Dehydrogenation Studies

The hydrogen storage properties of the as-synthesized alloy were further studied by the volumetric Sieverts‘ apparatus. Between each hydrogenation process, the sample was degassed at 400 °C (the highest operational temperature available for the used apparatus). The preliminary TG/DSC/MS studies proved that prolonged dehydrogenation at 400 °C is sufficient to finish the γ → β dehydrogenation reaction (Figure S17). Compared to TG/DSC/MS, the dehydrogenation process in Sieverts‘ apparatus is facilitated by the use of a dynamic vacuum. Therefore, all of the results of these hydrogenation experiments (with the exception of the first kinetic curve; see below) are related to the β → γ reactions.

**Figure 4:**
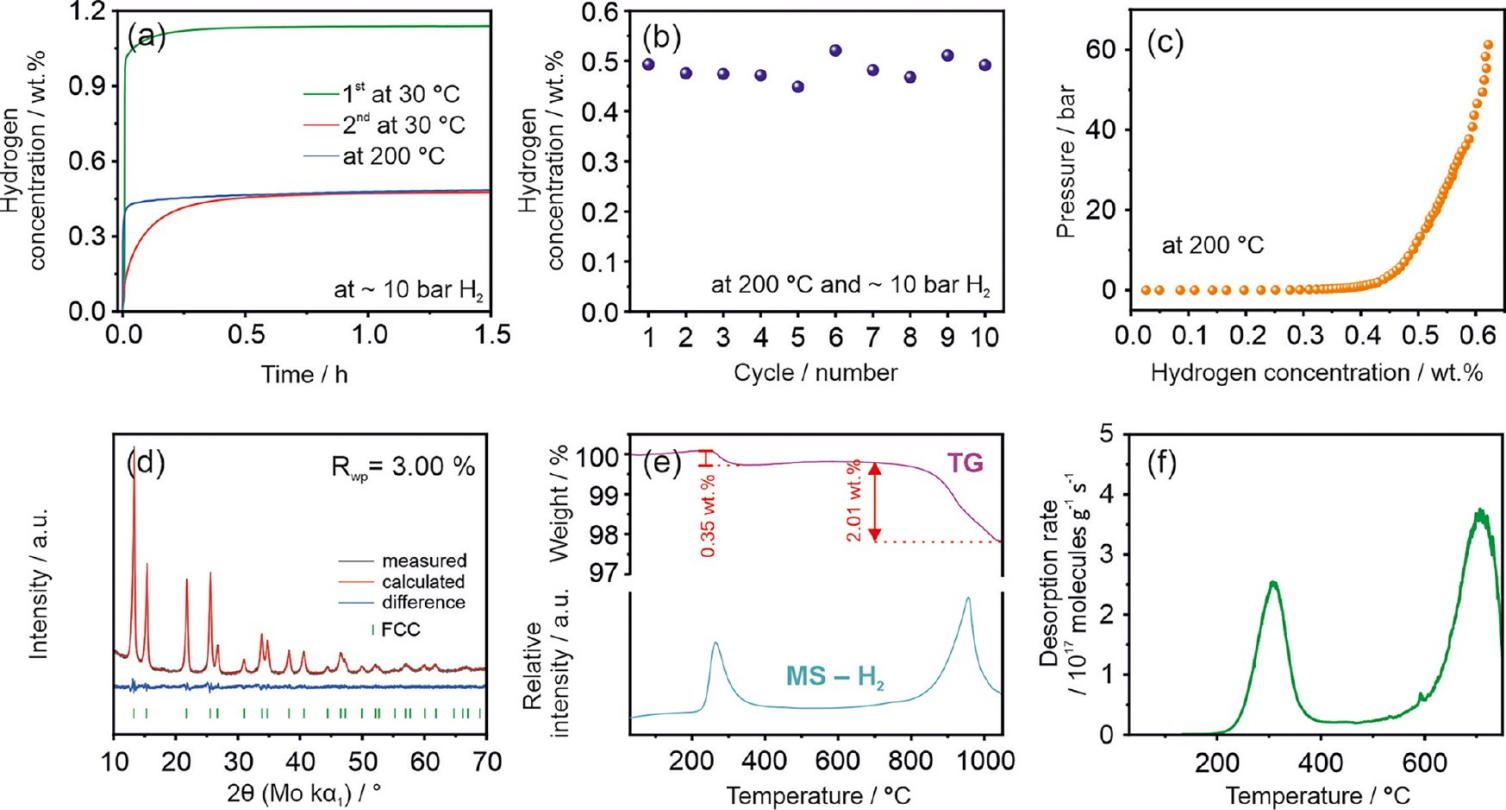
(a) Time–capacity (kinetic) curves obtained during hydrogenation of as-synthesized alloy at 30 and 200 °C using Sieverts‘ apparatus (the starting pressure was close to 10 bar H_2_). (b) Hydrogen absorption capacity during 10 cycles of absorption/desorption studies at 200 °C and the starting pressure close to 10 bar H_2_, (c) PCI obtained at 200 °C, (d) XRD pattern and its Pawley fit, (e) results of TG, and MS (H_2_) studies (5 °C/min), and (f) TDS spectrum (0.6 °C/min). XRD, TG/MS, and TDS were measured on the hydrogenated sample after a series of experiments using Sieverts‘ apparatus.

At first, the hydrogen uptake of the as-synthesized alloy was studied in a series of kinetic experiments (Figure 4a). The first kinetic curve obtained at 30 °C revealed further interesting features of the alloy. The material absorbs 1.15 wt % H_2_ within a fast reaction completed within 20 min (Figure 4a). Due to the fast reaction rate, the intermediate hydrogenation steps (visible in Figure 2a) were not observed. The process is not characterized by any incubation time. Moreover, it does not require any activation process, unlike the sample in the HPDSC experiments (Figure 3a). The need (or lack thereof) for activation results from different handling of the sample in both experiments and is directly related to the surface oxidation of the sample, which is excluded in the volumetric experiments.

After the dehydrogenation process (described above), the second hydrogenation kinetic experiment at 30 °C shows that the sample absorbed ∼0.45 wt % H_2_ within 30 min reaching finally ∼0.5 wt % H_2_ after 1.5 h. The difference between the hydrogen storage capacity reached in the first and second kinetic experiments at 30 °C should be sought in the presented TG curve obtained for the material after the first hydrogenation (Figure 2b). During the first contact with hydrogen, the as-synthesized sample absorbs it in the form of a highly stable β phase followed by the formation of the γ phase. Throughout the desorption to 400 °C, only the hydrogen atoms connected to the γ phase are desorbed. All of the hydrogen atoms bonded to the alloy in the form of the β phase will not be desorbed under these conditions. Therefore, in the second kinetic experiment at 30 °C, the material absorbs significantly less hydrogen. Moreover, the hydrogenation reaction rate is comparably slower in the second kinetic experiment at 30 °C, which can be indirectly explained by the different binding energies (in hydrogen–metal bond) of hydrogen atoms being stored in the β and γ structures (see the detailed discussion of DFT results). As a result, the formation of the β phase is associated with faster kinetics and greater hydrogen uptake compared to the formation of the γ phase.

By increasing the temperature to 200 °C, the β → γ hydrogenation kinetics can be significantly improved without the simultaneous destabilization of the hydride that could cause a decrease in capacity (Figure 4a). In this case, most of the hydrogen is absorbed within the first 10 min. At 200 °C, the hydrogen storage capacity related to the β → γ reaction is maintained over cycling at ∼0.5 wt % (Figure 4b), which indicates the stability of the material. The PCI related to the β → γ hydrogenation shows that the plateau pressure is lower than 0.5 bar of H_2_ (Figure 4c). In most of the plateau region, the pressure is below the detection limit of the used apparatus, which is undoubtedly related to high material affinity toward hydrogenation. The sloped saturation region follows the plateau region. The slope and associated differences in the storage capacities for different absorption pressures are responsible for the slight capacity deviation visible in Figure 4b.

After a series of experiments using a Sieverts’ apparatus, the ScYNdGd alloy in the fully hydrogenated state has been further investigated in detail to determine its structure, microstructure, and dehydrogenation process. The XRD pattern proves that hydrogen stabilized a single-phase FCC solid solution structure with a lattice parameter equal to 5.3036(2) Å (Figure 4d). Moreover, the cyclic hydrogenation/dehydrogenation experiments led to the formation of an alloy with a higher crystallinity (the reflections are better defined compared to the one-time hydrogenated sample; Figure S18). It should be related to the progressive crystalline size homogenization and growth during cycling, which can be, respectively, connected with a greater amount of absorbed hydrogen (as discussed below) and the applied hydrogenation/dehydrogenation temperatures. The latter may be explained as follows: the greater the hydrogen concentration, the more interstitial sites are occupied, and the distribution of hydrogen atoms becomes more uniform, leading to a decrease in the lattice distortion and homogenization of crystal lattice parameter.

**Figure 5:**
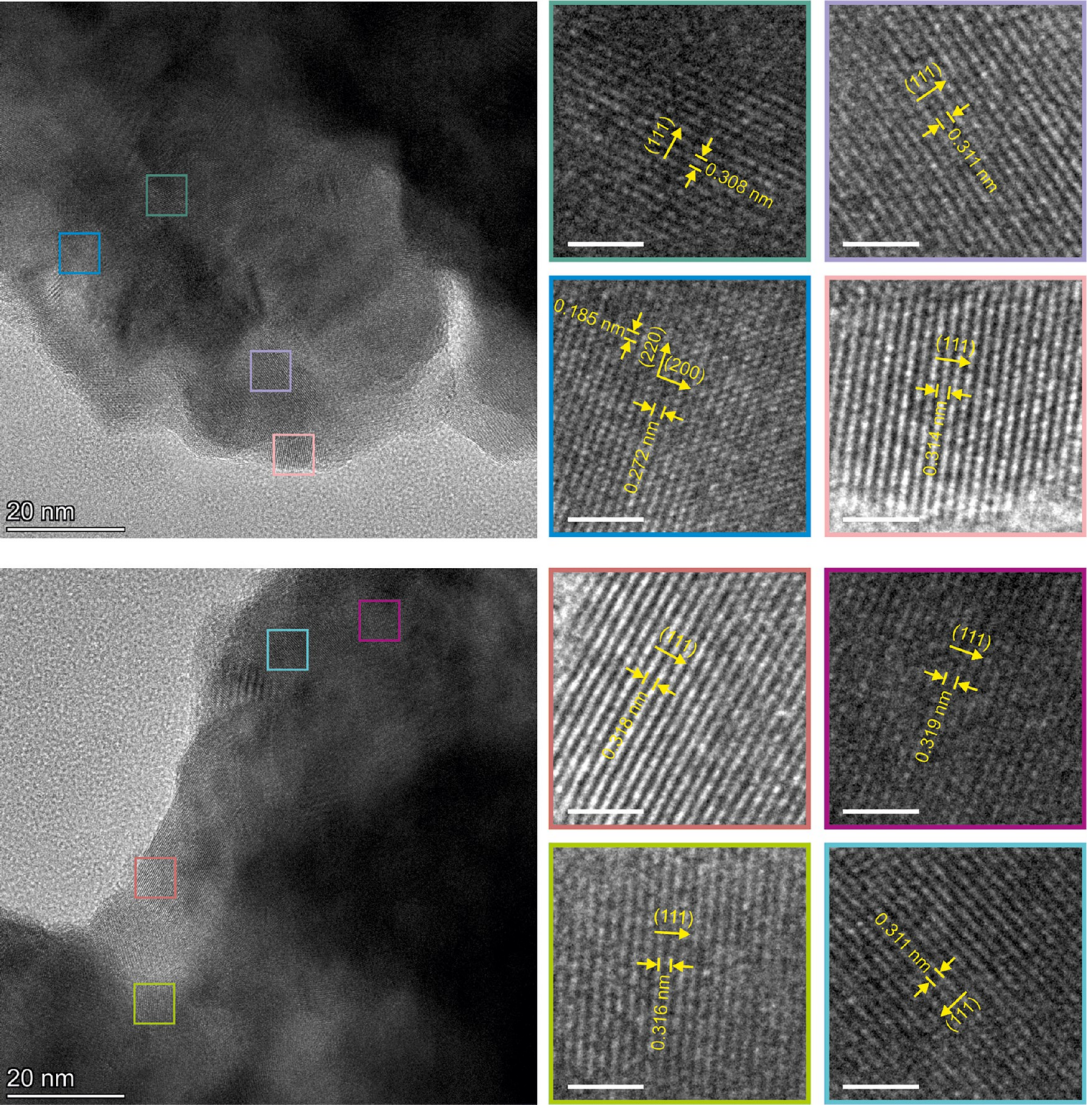
STEM micrographs obtained for hydrogenated ScYNdGd alloy after a series of experiments using Sieverts‘ apparatus (all at 200 kV). Scale bars in the magnified parts: 2 nm.

The improvement of the material’s crystallinity and partial defects healing caused by cycling hydrogenation/dehydrogenation experiments was proven also by STEM analysis; the poly nanocrystalline structure has been observed both on the surface and in the bulk of the alloy particles (Figure 5). The amorphous regions observed in the as-synthesized alloy, are no longer present on the surface. The measured *d*_hkl_ interplanar spacing values fit well with those obtained from the Pawley fit (Figures 5, S19, and S20).

On a microscale, the cycled hydrogenation/dehydrogenation process led to slight pulverization with a limited reduction of particle sizes and locally visible particle fracturing caused by repeated expansion and contraction of the material’s cell volume (Figure S21). In contrast to the as-synthesized sample, EDX analysis of the cycled sample shows the uniform distribution of the elements on the particle scale, proving the formation of a single-phase material (Figure S22). The only local chemical inhomogeneity was detected for Fe impurities (<0.5 atomic %) originating from the abrasion of the milling media.

The dehydrogenation of the fully hydrogenated cycled sample was studied by a number of complementary techniques. TG curve showed an overall decrease of capacity of 2.36 wt %, which corresponds to 2.5 H/M (Figure 4e). Such high H/M has only been once reported for MEAs and HEAs (for TiVZrNbHf),^9^ for which H/M is usually limited to 2.0 for the BCC solid solution alloy. The hydrogen storage capacity of ScYNdGd alloy could be even higher than measured due to the detrimental effect of oxidation on the measured weight loss (Chapter 6 in the Supporting Information).

The amount of hydrogen desorbed within the first dehydrogenation step (γ → β) is comparable to that measured after one hydrogenation and is equal to 0.35 wt % H_2_ (Figures 2b and 4e). This amount is, however, slightly lower (by 0.15 wt %) than the absorption capacity measured in the kinetic experiments at 200 °C (Figure 4b). There are several explanations for this phenomenon: (a) the counteracting effect of oxidation (Chapter 6 in the Supporting Information), which does not occur in the volumetric experiments; (b) the partial instability of the γ phase: during the handling of the hydrogenated sample, a part of the absorbed hydrogen is desorbed before starting the TG experiment. It is indicated by the TG results of the RMA sample (Figure S12b) and reported partial desorption of hydrogen from some REE (like Sc) at atmospheric pressure;^37^ (c) the additional 0.15 wt % is stored not in the γ but as the extended β phase. The slightly lower hydrogen concentration stored in the γ phase may simultaneously result from all three phenomena.

In the case of the second dehydrogenation step (β → α → M) the measured amount of desorbed hydrogen is significantly larger (2.01 wt %) than measured after one hydrogenation process, 1.07 wt % (Figures 2b and 4e). The highly increased hydrogen concentration observed for the β phase is also suggested by the intense MS signal observed for this part of hydride decomposition (Figure 4e). The larger storage capacity after cycling at least partly results from the observed improvement of the alloy’s crystallinity and defects healing; the fewer structural defects in the alloy, the more interstitial sites for hydrogen storage. Moreover, it may also be related to the mechanism of γ phase formation, which must first take place on the particle surface and can act as a layer, blocking further hydrogen diffusion. Once the γ → β dehydrogenation occurs, the next portion of hydrogen can diffuse to the inner part of the alloy and be stored as the β phase before the blocking γ phase is formed again on the surface. This explanation fits well with the discussion of the discrepancy in the storage capacities measured in TG and kinetic experiments.

The fully hydrogenated sample was also tested by TDS operating under a dynamic vacuum to demonstrate that the desorption conditions had a significant effect on the dehydrogenation process. The obtained spectrum proved the two-step desorption process in ScYNdGd hydride (Figure 4f). Moreover, the TDS spectrum revealed that differently than in the case of the TG curve, the dehydrogenation under vacuum is a continuous process with the release of hydrogen also taking place between the main two decomposition reactions. The similar gradual liberation of H atoms between the mentioned decomposition peaks was observed in the past for the Sc–H system.^37^ The comparison of the TDS spectrum (performed under dynamic vacuum with a slow heating rate of 0.6 °C/min) with the TG curve (performed under Ar flow with a heating rate of 5 °C/min) indicates the parameters insensitivity of the γ → β dehydrogenation process (taking place between 200 and 400 °C in both cases) and a large desorption parameters dependence on the β phase decomposition temperature (in the case of TDS, the process starting temperature is decreased by about 200 °C). The desorption peak temperatures measured by TDS (∼310 and 710 °C) are comparable to those measured in the past for binary REE-H systems.^33^

**Figure 6:**
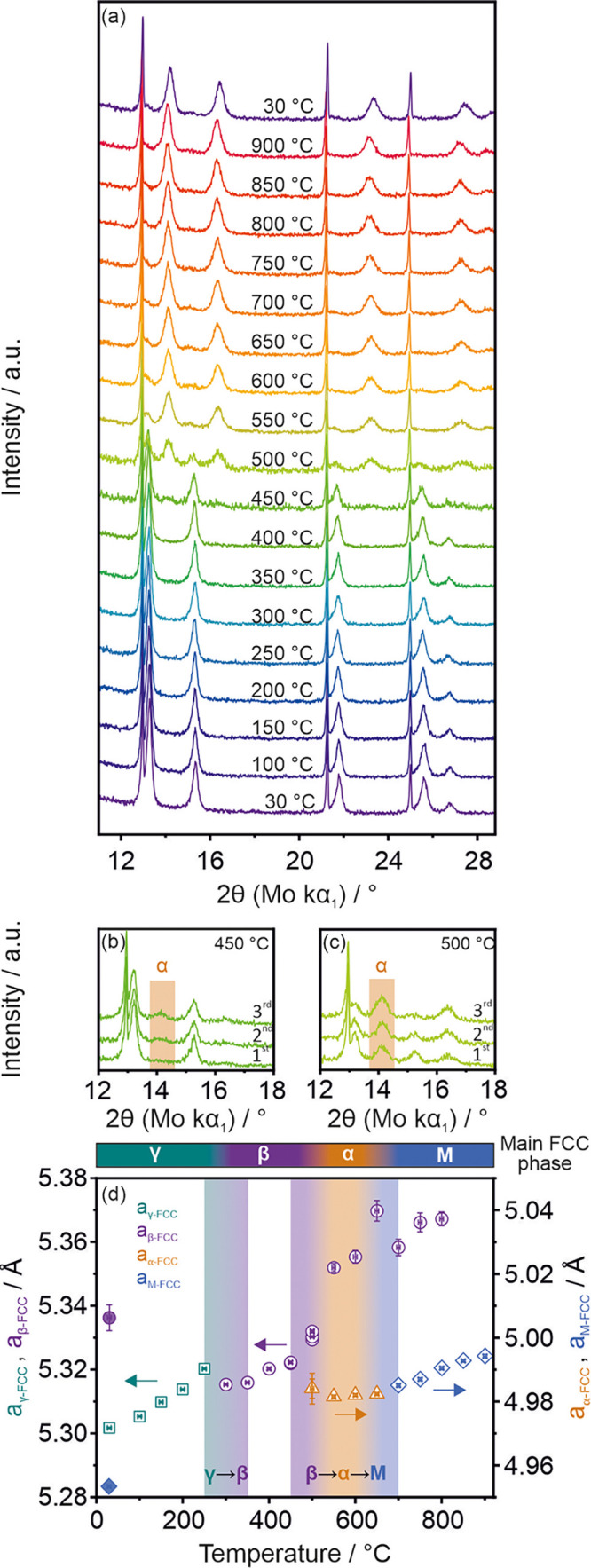
(a) XRD patterns of the fully hydrogenated sample (after a series of experiments using Sieverts‘ apparatus) obtained during the in situ heating experiment (from 30 to 900 °C and after cooling to 30 °C). Each pattern is the average of three consecutive measurements during which no structure change was observed (except at 450 and 500 °C). The reflections visible at around 12.97, 21.26, and 24.99° 2θ (Mo kα_1_) correspond to the Si NISTe standard added to the system to monitor the temperature of the sample. (b, c) Three consecutive XRD patterns obtained (during the in situ experiment) at 450 and 500 °C, respectively. The highlighted area points out the 2θ range of the main reflection of the newly formed α-FCC phase. (d) Lattice parameters (from Pawley fits) of the FCC phases observed at different temperatures during the in situ experiment. The highlighted areas indicate the temperature ranges at which the dehydrogenation steps appeared. The filled symbols correspond to the lattice parameter values of the alloy cooled to 30 °C.

The structural changes during the dehydrogenation process of the fully hydrogenated alloy were followed by in situ XRD. Figure 6a shows the XRD patterns collected at every 50 °C during heating from 30 to 900 °C. The patterns are averages of three consecutive measurements at the same temperature. The only exceptions are the patterns obtained at 450 and 500 °C for which an evolution of the structure during isothermal heating was observed (Figure 6b,c). The lattice parameters of the detected phases are listed in Figure 6d. The increase and decrease of the lattice parameters, visible in Figure 6d, are caused by thermal expansion of the material and hydrogen desorption, respectively.

The initial single-phase FCC solid solution structure of fully hydrogenated alloy (from now on, for clarity, named γ-FCC) is maintained up to 250 °C. At this temperature, the γ → β dehydrogenation process starts associated with the decrease of the lattice cell volume without a phase transition process. The γ → β process occurred at the same temperature range as in the case of the TG and TDS experiments. Between 350 and 450 °C the alloy exists as the β-FCC phase. The maintenance of FCC structure during the dehydrogenation of the γ phase follows the trend of some RE elements such as Ce, Pr, or Nd.^33^

The second dehydrogenation process (β → α → M), which ends in the metallic state of the alloy, starts at 450 °C. At this temperature, a new FCC phase with a significantly lower lattice parameter is formed (Figure 6b,c). The newly formed phase should be first assigned to the α phase (α-FCC) and in the final stage at 700 °C to the alloy’s metallic state (M-FCC). While the increase in the temperature progresses, the α-FCC phase replaces the β-FCC phase. The process continues until at least 700 °C, as indicated by the relatively stable lattice parameter of the α-FCC phase. This unusual thermal behavior results from two counteracting processes: thermal expansion of the compound and simultaneous dehydrogenation. The temperature of β → α → M processes is lower than the one observed in TG or TDS experiments, proving that the second dehydrogenation process temperature is highly dependent on the desorption parameters (among other atmosphere pressure, sample placements, heating systems). Overall, the dehydrogenation process that takes place within the studied REE-MEA structure can be described as γ-FCC → β-FCC → α-FCC → M-FCC. The absence of phase transformations upon hydrogenation from the metallic state to γ suggests that the studied ScYNdGd alloy mimics the properties of the Ce–H system for which an absence of phase transition was reported.^36^

The rapid increase in the lattice parameter of the residual β-FCC phase above 450 °C suggests changes in the chemical composition of this phase (Figure 6d). Most probably, during the β → α reaction, partial chemical segregation occurs. The large lattice parameter of the β-FCC phase indicates that above 450 °C it is composed mainly of relatively large atoms, which, in this study, are Gd and Nd. These two elements, at the same time, form more stable hydrides than Sc and Y. This means that in the ScYNdGd alloy, the hydrogen atoms are the latest desorbed from the interstitial sites mostly surrounded by Gd or Nd atoms. Finally, the remains of the β-FCC phase are still visible even at 900 °C–this means that the dehydrogenation process is not entirely finished even at this high temperature.

After heating to 900 °C, the sample has been cooled to 30 °C, unraveling the stability of the newly formed dehydrogenated M-FCC structure (Figure 6a,d). The lattice parameter of the metallic ScYNdGd phase equals 4.9533(5) Å. The presented data shows that the hydrogen-stabilized alloy retained a well-defined crystal structure after dehydrogenation–the alloy did not decompose to the original multiphase structure of the as-synthesized alloy. The absence of phase transformation during hydrogenation/dehydrogenation cycling explains well the discussed absence of severe pulverization of the material after cycling.

The dehydrogenation led to a reduction of the cell volume from 149.18(2) to 121.53(4) Å^3^. Considering that the approximate dissolution of hydrogen atoms leads to an expansion of the host metal lattice of 2.5(5) Å^3^, the studied hydride absorbed 2.77(55) H/M, which agrees with the other measured uptake values.^38^ Moreover, the calculated relative volume expansion (expansion per metal atom) reached 22.75%. Assuming that the H/M ratio of the final hydride composition is 2.5, it means that the relative expansion per one hydrogen atom in the structure is equal to 9.1%. This is, compared to other metal hydrides for which the volume expands by an order of 20% (also reaching up to 30% for the Mg–H system) very little, indicating (from an engineering point of view) good applicability of this alloy–the smaller the volume change of the material caused by hydrogenation/dehydrogenation processes the smaller the necessary dead volume in the storage tank.^34^

### DFT Studies

**Figure 7:**
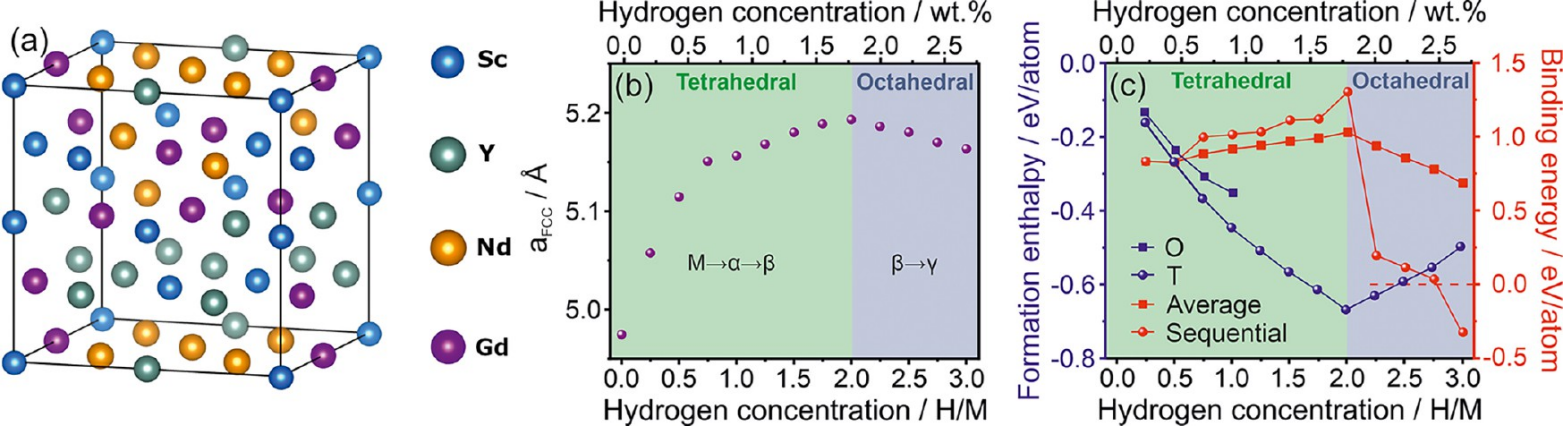
(a) Schematic view of the FCC ScYNdGd MEA; (b) calculated lattice parameter as a function of H/M ratio in ScYNdGd MEA; (c) the calculated formation enthalpy and binding energy (average and sequential) as a function of H/M ratio in ScYNdGd MEA. The symbols O and T stand for the calculated energies considering the preferential occupation of octahedral or tetrahedral interstitial sites, respectively. The dashed red line differentiates the regions with positive and negative binding energies.

DFT calculations provided further insight into the hydrogenation/dehydrogenation process. Figure 7a shows the schematic view of the structural model (FCC cell randomly built by Sc, Y, Gd, and Nd) that was used for the calculations. Figure 7b presents the calculated FCC lattice parameter’s change to the structure’s hydrogen concentration. The calculated lattice parameter of the alloy in its metallic state equals 4.97 Å, which is in good agreement with the experimental data (4.9533(5) Å measured for the sample cooled after in situ XRD experiments). The calculations predicted cell volume expansion during progressing hydrogenation of the alloy up to the level of the fully hydrogenated β phase. However, the lattice parameter calculated for alloy hydride with a H/M ratio of 2.5 (5.18 Å) differs from that measured at 30 °C for a fully hydrogenated sample (5.3037(2) Å). The calculations also suggest a contraction of the lattice cell during further hydrogenation of β to γ phase, which is typical for Ce–H or Nd–H systems but has not been observed experimentally in this work.^36,39^

According to the Switendick criterion, the H–H distance in hydrides should be larger than 2.1 Å due to the repulsive H–H interactions.^40^ In the FCC structure, the distance between the nearest pair of tetrahedral (T–T) sites and tetrahedral-octahedral (T–O) sites equals 0.5a and 0.433a, respectively (a is a lattice parameter of hydride). Based on the measured lattice parameter of the fully hydrogenated ScYNdGd-based hydride (5.3036(2) Å), the T–T and T–O sites distances are 2.652 and 2.296 Å, indicating possible occupation of both T and O sites by hydrogen atoms. Therefore, we calculated the relevant formation enthalpies (Δ*H*_f_) in the low-hydrogen-concentration region to see whether the hydrogen atoms tend to occupy O or T sites first (Figure 7c). All of the calculated formation enthalpy values are negative, meaning that the hydrogenation process is an exothermic reaction. The DFT studies, which evaluate the stability of MEA hydride with partially filled T or O sites, unequivocally indicate that hydrogen fills first the energetically favorable tetrahedral interstitial sites. All available tetrahedral interstitial sites are filled with the final saturation of the β phase. The further hydrogenation of the material to form the γ phase is associated with the filling of the octahedral sites. This behavior shows that the ScYNdGd alloy mimics the hydrogenation behavior of the REE dihydrides of FCC structure, such as Ce.^36,39^

The calculated binding energies (that evaluates the interaction between hydrogen and metal atoms in MEA), both average and sequential, show that up to the complete hydrogenation of the β phase (H/M = 2), each successive hydrogen atom is bound more and more strongly in the alloy structure; more positive binding energy indicates the stronger interaction between hydrogen and MEA. It agrees with the great proneness of the ScYNdGd alloy to form a β phase (manifested through fast hydrogenation kinetics at 30 °C; Figure 4a). Moreover, it has been experimentally proven by the TG curves of hydride decomposition that the more hydrogen is stored in the β phase, the higher the final hydride decomposition temperature (Figures 2b and 4e).

The trend is the opposite in the case of the γ phase formation. The binding energies are drastically smaller for the hydrogen atoms stored in the O sites compared to those stored in the T sites. Moreover, the binding energy tends to significantly decrease with the increase of the hydrogen atom concentration in the range of H/M between 2.0 and 3.0. It explains well the lower thermal stability of the γ phase (in comparison to the β phase). A similar trend was observed for Ti–V–Nb–Zr–Ta BCC HEA, where the occupation of the T sites resulted in enhanced thermal stability while the filling of O sites in the destabilization of created hydride.^15^ However, this is not a general tendency in MEAs or HEAs but rather a dependence related to the type of solid solution structure and elastic or chemical effects.^16^ Interestingly, in the studied ScYNdGd hydride, the calculated sequential binding energies become negative in the region of H/M equal to 2.75, pointing to the maximal hydrogen storage capacity of the system around this value. It is consistent with the experimental observations, which show a maximum storage capacity of 2.5 H/M.

## Conclusions and Outlook

This study is devoted to REE-MEA, which is characterized by intriguing and unprecedented hydrogen storage properties. In summary, we demonstrated that the single-phase structure of nanocrystalline MEA ScYNdGd can be stabilized to a great extent by hydrogenation. Ex situ and in situ XRD studies proved the high stability of the obtained FCC solid solution structure at different hydrogen concentrations. Therefore, MA and cyclic hydrogenation/dehydrogenation reactions should be considered as promising approaches for the synthesis and stabilization of MEAs and HEAs. Moreover, the detailed hydrogenation/dehydrogenation studies and DFT calculations revealed hydride formation with remarkably high storage capacity, reaching at least 2.5 H/M. Hydrogenation occurs at very low pressures and with fast process kinetics, showing stable hydrogen storage capacity related to the γ phase formation/decomposition process. When stored under oxidation-preventing/inert conditions, the alloy does not require any activation prior to its hydrogenation. Moreover, when it is surface oxidized, the activation process is simple, requiring only one cycle of hydrogenation/dehydrogenation to activate the alloy for hydrogenation at room temperature, even after months of the oxidation process. Unusually for metal hydrides with a high hydrogen storage capacity, the alloy goes through successive stages of dehydrogenation without a phase transition, preserving the FCC structure (imitating the Ce–H system). The results of complementary measurement techniques are consistent and show that the desorption process proceeds in two steps. First, a relatively low-temperature process (200–400 °C) of γ-phase decomposition occurs in which hydrogen atoms are desorbed from O sites. Second, the high-temperature process, in which desorption conditions can moderate the reaction temperature, is related to the decomposition of the β phase into the alloy in its metallic state. As DFT calculations showed, the hydrogen atoms that form the β phase are stored in T sites and are characterized by a binding energy increase with greater hydrogen content.

We think that the presented research can be a starting point for further research, for example, focusing on a detailed understanding of hydrogen-induced stabilization effect, outstanding activation properties, hydrogen storage behavior of FCC MEAs and HEAs in general, or on achieving H/M as great as 3 with new REE-based MEAs or HEAs (the capacity limit for REE-H systems). Moreover, further research on REE-based MEAs or HEAs could also focus on the optimization of their hydrogen storage properties by using strategies already successfully applied in the BCC HEAs and C14 Laves multi-principal element alloys: reduction of hydride stability by co-alloying with hydride nonforming elements,^17,18^ increase of the gravimetric hydrogen storage capacity by the reduction of the molar mass of the alloy by co-alloying with lightweight elements,^19−21^ designing the alloys by thermodynamic modeling.^23−25^ Furthermore, co-alloying with cheaper elements can be a possible strategy to reduce the overall price of the alloy.

The motivation for undertaking this study was strongly related to basic research; however, the presented findings are also of an applied nature. The successful synthesis of REE-MEAs with FCC structure extends the scope of the possible research to the applications known for the binary REE-H. Moreover, the development of new alloys/hydrides for these applications can significantly benefit from HEAs’ adjustable and vast concept.^10^ For example, as Y exhibits high hydrogen storage density, low hydrogen desorption pressure, and high-temperature stability, it is considered a solid-state neutron moderator in compact nuclear reactors (self-regulating, truck-transportable reactor with 1 kWe to 10 MWe electric power output)–weakening the neutron energy spectrum.^41^ The applicability of metal hydrides in this sector is heavily limited by hydrogenation-/dehydrogenation-induced crack formation (due to the phase transformations) and brittleness of the hydride phase that threatens the integrity of the hydride. In this context, the studied ScYNdGd alloy with the lack of hydrogen-induced phase transformation and mild volume expansion gives promise of crack-free MEAs or HEAs.

Moreover, most REEs undergo a phase transition during hydrogenation, and the same is expected for some of the REE-based MEAs and HEAs. The phase change is often associated with optical, electrical, or magnetic switchable properties. Some REEs show the reversible metal–insulator transition upon hydrogenation (between 2 and 3 H/M). It is correlated with a change of the optical properties: conductive and reflective alloys transform to insulating and transparent (optical gaps on the order of 2 eV) at high H concentrations. The fact that the reversible transition takes place at room temperature and is very smooth allows their application as adjustable and switchable mirrors or coatings, optical switches, and eye-visible hydrogen sensing systems.^42,43^ The phase transformation can also induce switchable antiferromagnetic to ferromagnetic properties.^32^ Finally, the REE-based binary and ternary hydrides exhibit superconductive properties under elevated pressures, which should also be expected for some MEAs and HEAs.^44,45^

## Supporting Information Available

Methodology; characterization of the substrates (XRD, TG, MS); additional XRD patterns and their Pawley fits obtained at different stages of the synthesis process; SEM, STEM, and EDX characterization of as-synthesized and fully hydrogenated alloy; HPDSC experiments under different H_2_ pressures; repeatability of HPDSC under 15 bar H_2_; complementary TG/DSC dehydrogenation process data; evolution of structure and storage properties during RMA; and detailed results of oxidation and activation/reactivation experiments (PDF)

## References

[ref1] Yeh J.-W.; Chen S.-K.; Lin S.-J.; Gan J.-Y.; Chin T.-S.; Shun T.-T.; Tsau C.-H.; Chang S.-Y. Nanostructured high-entropy alloys with multiple principal elements: novel alloy design concepts and outcomes. Adv. Eng. Mater. 2004, 6, 299–303. 10.1002/adem.200300567.

[ref2] Zhai Y.; Ren X.; Wang B.; Liu S. High-entropy catalyst—a novel platform for electrochemical water splitting. Adv. Funct. Mater. 2022, 32, 2207536 10.1002/adfm.202207536.

[ref3] Zhang Y. High-Entropy Materials Advances and Applications, 1st ed.; CRC Press, 2023.

[ref4] Amiri A.; Shahbazian-Yassar R. Recent progress of high-entropy materials for energy storage and conversion. J. Mater. Chem. A 2021, 9, 782–823. 10.1039/D0TA09578H.

[ref5] Zhang Y.; Wang D.; Wang S. High-entropy alloys for electrocatalysis: design, characterization, and applications. Small 2022, 18, 2104339 10.1002/smll.202104339. 34741405

[ref6] Yao Y.; Dong Q.; Brozena A.; Luo J.; Miao J.; Chi M.; Wang C.; Kevrekidis I. G.; Ren Z. J.; Greeley J.; Wang G.; Anapolsky A.; Hu L. High-entropy nanoparticles: Synthesis-structure-property relationships and data-driven discovery. Science 2022, 376, 151 10.1126/science.abn3103. 35389801

[ref7] Li H.; Lai J.; Li Z.; Wang L. Multi-sites electrocatalysis in high-entropy alloys. Adv. Funct. Mater. 2021, 31, 2106715 10.1002/adfm.202106715.

[ref8] Löffler T.; Ludwig A.; Rossmeisl J.; Schuhmann W. What makes high-entropy alloys exceptional electrocatalysts?. Angew. Chem., Int. Ed. 2021, 60, 26894–26903. 10.1002/anie.202109212. PMC9292432 34436810

[ref9] Sahlberg M.; Karlsson D.; Zlotea C.; Jansson U. Superior hydrogen storage in high entropy alloys. Sci. Rep. 2016, 6, 36770 10.1038/srep36770. 27829659 PMC5103184

[ref10] Marques F.; Balcerzak M.; Winkelmann F.; Zepon G.; Felderhoff M. Review and outlook on high-entropy alloys for hydrogen storage. Energy Environ. Sci. 2021, 14, 5191–5227. 10.1039/D1EE01543E.

[ref11] Karlsson D.; Ek G.; Cedervall J.; Zlotea C.; Møller K. T.; Hansen T. C.; Bednarčík J.; Paskevicius M.; Sørby M. H.; Jensen T. R.; Jansson U.; Sahlberg M. Structure and hydrogenation properties of a HfNbTiVZr high-entropy alloy. Inorg. Chem. 2018, 57, 2103–2110. 10.1021/acs.inorgchem.7b03004. 29389120

[ref12] Nygård M. M.; Ek G.; Karlsson D.; Sahlberg M.; Sørby M. H.; Hauback B. C. Hydrogen storage in high-entropy alloys with varying degree of local lattice strain. Int. J. Hydrogen Energy 2019, 44, 29140–29249. 10.1016/j.ijhydene.2019.03.223.

[ref13] Nygård M. M.; Sławiński W. A.; Ek G.; Sørby M.; Sahlberg M.; Keen D. A.; Hauback B. C. Local order in high-entropy alloys and associated deuterides – a total scattering and Reverse Monte Carlo study. Acta Mater. 2020, 199, 504–513. 10.1016/j.actamat.2020.08.045.

[ref14] Nygård M. M.; Ek G.; Karlsson D.; Sørby M. H.; Sahlberg M.; Hauback B. C. Counting electrons - A new approach to tailor the hydrogen sorption properties of high-entropy alloys. Acta Mater. 2019, 175, 121–129. 10.1016/j.actamat.2019.06.002.

[ref15] Hu J.; Zhang J.; Li M.; Zhang S.; Xiao H.; Xie L.; Sun G.; Shen H.; Zhou X.; Li X.; Li P.; Zhang J.; Vitos L.; Zu X. The origin of anomalous hydrogen occupation in high entropy alloys. J. Mater. Chem. A 2022, 10, 7228–7237. 10.1039/D1TA10649J.

[ref16] Hu J.; Zhang J.; Xiao H.; Xie L.; Sun G.; Shen H.; Li P.; Zhang J.; Zu X. A first-principles study of hydrogen storage of high entropy alloy TiZrVMoNb. Int. J. Hydrogen Energy 2021, 46, 21050–21058. 10.1016/j.ijhydene.2021.03.200.

[ref17] Ek G.; Nygård M. M.; Pavan A. F.; Montero J.; Henry P. F.; Sørby M. H.; Witman M.; Stavila V.; Zlotea C.; Hauback B. C.; Sahlberg M. Elucidating the effects of the composition on hydrogen sorption in TiVZrNbHf-based high-entropy alloys. Inorg. Chem. 2021, 60, 1124–1132. 10.1021/acs.inorgchem.0c03270. 33370527 PMC7871323

[ref18] Zlotea C.; Bouzidi A.; Montero J.; Ek G.; Sahlberg M. Compositional effects on the hydrogen storage properties in a series of refractory high entropy alloys. Front. Energy Res. 2022, 10, 991447 10.3389/fenrg.2022.991447.

[ref19] Montero J.; Ek G.; Sahlberg M.; Zlotea C. Improving the hydrogen cycling properties by Mg addit0ion in Ti-V-Zr-Nb refractory high entropy alloy. Scr. Mater. 2021, 194, 113699 10.1016/j.scriptamat.2020.113699.

[ref20] Strozi R. B.; Leiva D. R.; Huot J.; Botta W. J.; Zepon G. An approach to design single BCC Mg-containing high entropy alloys for hydrogen storage applications. Int. J. Hydrogen Energy 2021, 46, 25555–25561. 10.1016/j.ijhydene.2021.05.087.

[ref21] Strozi R. B.; Leiva D. R.; Huot J.; Botta W. J.; Zepon G. Synthesis and hydrogen storage behavior of Mg–V–Al–Cr–Ni high entropy alloys. Int. J. Hydrogen Energy 2021, 46, 2351–2361. 10.1016/j.ijhydene.2020.10.106.

[ref22] Witman M.; Ek G.; Ling S.; Chames J.; Agarwal S.; Wong J.; Allendorf M. D.; Sahlberg M.; Stavila V. Data-driven discovery and synthesis of high entropy alloy hydrides with targeted thermodynamic stability. Chem. Mater. 2021, 33, 4067–4076. 10.1021/acs.chemmater.1c00647.

[ref23] Zepon G.; Silva B. H.; Zlotea C.; Botta W. J.; Champion Y. Thermodynamic modelling of hydrogen-multicomponent alloy systems: Calculating pressure-composition-temperature diagrams. Acta Mater. 2021, 215, 117070 10.1016/j.actamat.2021.117070.

[ref24] Strozi R. B.; Silva B. H.; Leiva D. R.; Zlotea C.; Botta W. J.; Zepon G. Tuning the hydrogen storage properties of Ti-V-Nb-Cr alloys by controlling the Cr/(TiVNb) ratio. J. Alloys Compd. 2023, 932, 167609 10.1016/j.jallcom.2022.167609.

[ref25] Hu J.; Zhang J.; Xiao H.; Xie L.; Shen H.; Li P.; Zhang J.; Gong H.; Zu X. A density functional theory study of the hydrogen absorption in high entropy alloy TiZrHfMoNb. Inorg. Chem. 2020, 59, 9774–9782. 10.1021/acs.inorgchem.0c00989. 32589411

[ref26] Kumar A.; Yadav T. P.; Mukhopadhyay N. K. Notable hydrogen storage in Ti–Zr–V–Cr–Ni high entropy alloy. Int. J. Hydrogen Energy 2022, 47, 22893–22900. 10.1016/j.ijhydene.2022.05.107.

[ref27] Balcerzak M.; Ternieden J.; Felderhoff M. Synthesis, thermal stability, and hydrogen storage properties of poorly crystalline TiVFeCuNb multi-principal element alloy. J. Alloys Compd. 2023, 943, 169142 10.1016/j.jallcom.2023.169142.

[ref28] Ponsoni J. B.; Balcerzak M.; Botta W. J.; Felderhoff M.; Zepon G. A comprehensive investigation of the (Ti_0.5_Zr_0.5_)_1_(Fe_0.33_Mn_0.33_Cr_0.33_)_2_ multicomponent alloy for room-temperature hydrogen storage designed by computational thermodynamic tools. J. Mater. Chem. A 2023, 11, 14108–14118. 10.1039/D3TA02197A.

[ref29] Mohammadi A.; Ikeda Y.; Edalati P.; Mito M.; Grabowski B.; Li H.-K.; Edalati K. High-entropy hydrides for fast and reversible hydrogen storage at room temperature: Binding-energy engineering via first-principles calculations and experiments. Acta Mater. 2022, 236, 118117 10.1016/j.actamat.2022.118117.

[ref30] Steurer W. Single-phase high-entropy alloys – A critical update. Mater. Charact. 2020, 162, 110179 10.1016/j.matchar.2020.110179.

[ref31] Ye Y. F.; Wang Q.; Lu J.; Liu C. T.; Yang Y. High-entropy alloy: challenges and prospects. Mater. Today 2016, 19, 349–362. 10.1016/j.mattod.2015.11.026.

[ref32] Kong B.; Zhang L.; Chen X.-R.; Deng M.-S.; Cai L.-C.; Ling-Hu R.-F. Magnetic, electronic and optical properties of lanthanide hydrides, GdH_2_ and GdH_3_. J. Phys. Chem. Solids 2013, 74, 1322–1328. 10.1016/j.jpcs.2013.04.012.

[ref33] Yartys V. A.; Gutfleisch O.; Panasyuk V. V.; Harris I. R. Desorption characteristics of rare earth (R) hydrides (R = Y, Ce, Pr, Nd, Sm, Gd and Tb) in relation to the HDDR behaviour of R–Fe-based-compounds. J. Alloys Compd. 1997, 253–254, 128–133. 10.1016/S0925-8388(96)03097-6.

[ref34] Peisl H. Lattice strains due to hydrogen in metals. In Hydrogen in Metals I. Topics in Applied Physics; Alefeld G.; Völkl J., Eds.; Springer: Berlin, Heidelberg, 1978; Vol. 28.

[ref35] Demchyna R. O.; Chykhrij S. I.; Kuz’ma Y. B. Y-Cu-P system. J. Alloys Compd. 2002, 345, 170–174. 10.1016/S0925-8388(02)00432-2.

[ref36] Priyanga G. S.; Rajeswarapalanichamy R.; Iyakutti K. First principles study of structural, electronic, elastic and magnetic properties of cerium and praseodymium hydrogen system REHx (RE: Ce, Pr and x = 2,3). J. Rare Earths 2015, 33, 289–303. 10.1016/S1002-0721(14)60417-8.

[ref37] Bashkin I. O.; Ponystovskii E. G.; Kost M. E. Phase transformations in hydrides of rare-earth metals under pressure. Phys. Status Solidi B 1977, 83 (2), 517–520. 10.1002/pssb.2220830219.

[ref38] Dornheim M. Thermodynamics of metal hydrides: Tailoring reaction enthalpies of hydrogen storage materials. In Thermodynamics - Interaction Studies - Solids, Liquids and Gases; InTech, 2011.

[ref39] Pebler A.; Wallace W. E. Crystal structures of some lanthanide hydrides. J. Phys. Chem. A 1962, 66, 148–151. 10.1021/j100807a033.

[ref40] Switendick A. C. Band Structure Calculations for Metal Hydrogen Systems. Z. Phys. Chem. 1979, 117, 89–112. 10.1524/zpch.1979.117.117.089.

[ref41] Peng J.; Wu M.; Du F.; Yang F.; Shen J.; Wang L.; Ye X.; Yan G. Thermodynamic modelling of YeH and YeZreH system aided by first-principles and its application in bulk hydride moderator fabrication. J. Nucl. Mater. 2020, 531, 152035 10.1016/j.jnucmat.2020.152035.

[ref42] Huiberts J. N.; Griessen R.; Rector J. H.; Wijngaarden R. J.; Dekker J. P.; de Groot D. G.; Koeman N. J. Yttrium and lanthanum hydride films with switchable optical properties. Nature 1996, 380, 231–234. 10.1038/380231a0.

[ref43] Ngene P.; Radeva T.; Slaman M.; Westerwaal R. J.; Schreuders H.; Dam B. Seeing hydrogen in colors: low-cost and highly sensitive eye readable hydrogen detectors. Adv. Funct. Mater. 2014, 24, 2374–2382. 10.1002/adfm.201303065.

[ref44] Zeng T.-X.; Kong B.; Diao X.-F. First principles studies of the structural stability and lattice dynamics for rare earth hydridies REH_x_ (RE = Sm, Gd, Tb, Dy, Ho, Tm, x = 2, 3) under pressure. J. Phys. Chem. Solids 2019, 126, 196–208. 10.1016/j.jpcs.2018.11.017.

[ref45] Chen W.; Huang X.; Semenok D. V.; Chen S.; Zhou D.; Zhang K.; Oganov A. R.; Cui T. Enhancement of superconducting properties in the La–Ce–H system at moderate pressures. Nat. Commun. 2023, 14, 2660 10.1038/s41467-023-38254-6. 37160883 PMC10170082

