# Supplementary material for: Hydrogen-Stabilized ScYNdGd Medium-Entropy Alloy for Hydrogen Storage
Source: J Am Chem Soc. 2024 Feb 14;146(8):5283–94. doi: 10.1021/jacs.3c11943 (PMC10910561; doi:10.1021/jacs.3c11943)
Supplement: Supplementary file 1 — ja3c11943_si_001.pdf [file ja3c11943_si_001.pdf]

SUPPORTING INFORMATION

It is Supporting Information data for the publication
'Hydrogen-Stabilized ScYNdGd Medium-Entropy Alloy for Hydrogen Storage'.

Mateusz Balcerzak[a,b,*], Jéssica Bruna Ponsoni[a,c], Hilke Petersen[a], César Menéndez[d], Jan Ternieden[a], Linda Zhang[e,f], Frederik Winkelmann[a], Kondo-Francois Aguey-Zinsou[d], Michael Hirscher[e,f], and Michael Felderhoff[a]

[a] Heterogeneous Catalysis Department, Max-Planck-Institut für Kohlenforschung, Kaiser-Wilhelm Platz 1, 45470, Mülheim an der Ruhr, Germany

[b] Institute of Materials Science and Engineering, Poznan University of Technology, Jana Pawła II No 24, Poznan, 61-138 Poland

[c] Graduate Program in Materials Science and Engineering (PPGCEM/UFSCar), Federal University of Sao Carlos, Rodovia Washington Luiz, km 235, São Carlos, São Paulo CEP, 13565-905, Brazil

[d] MERLin, School of Chemistry, University of Sydney, NSW 2006, Australia

[e] Max-Planck-Institut für Intelligente Systeme, Heisenbergstrasse 3, 70569, Stuttgart, Germany

[f] Advanced Institute for Materials Research, Tohoku University, Katahira 2-1-1, Aoba-ku, Sendai, 980-8577, Japan.

* E-mail: balcerzak@kofo.mpg.de

The SI was created to not only present but also discuss some of the supplementary research data. The file also contains a detailed description of experimental procedures. The data and descriptions were divided into seven subchapters.

Table of Contents

1. **Methodology** **S3**
2. **Starting materials** **S7**
3. **Semi-empirical parameters for the design of MEAs** **S8**
4. **Mechanochemical synthesis and thermal stability** **S9**
5. **First hydrogenation/dehydrogenation experiments** **S14**
6. **Oxidation, activation, and reactivation of alloy** **S18**
7. **Detailed hydrogenation/dehydrogenation studies** **S23**

References **S29**

1. Methodology

Starting materials. All the alloys discussed in this study were synthesized using pure Sc-H (Nano Research Elements, 99.9% < 45 μm), Y-H (ABCR, 99.9%, < 400 μm), Nd (Alfa Aesar, 99.8%, < 40 μm), and Gd-H (Alfa Aesar, 99.9%, < 200 μm). We motivate using three elements in the hydrogenated state (available in our laboratory) by increasing the brittle character of the milled materials, thus avoiding severe compensation of the milled material to the milled media (milling vial walls and balls). The crystal structure of all the starting materials and their hydrogen content were investigated prior to the synthesis of MEA by X-ray diffraction (XRD) and thermogravimetry/mass spectroscopy (TG/MS), respectively (Figure S1). The results of Rietveld refinement of the XRD data showed that in the case of all used hydrides, the main phase was MH_2 (Table S1).

Material preparation. To avoid oxidation, all the handling with materials before, during, and after synthesis was done in the MBrown glovebox filled with purified argon atmosphere (O_2 and H_2O < 1 ppm). The quaternary ScYNdGd alloy was synthesized via mechanical alloying (MA) as well as via reactive mechanical alloying (RMA). Both reactions were performed using planetary ball mills provided by Fritsch: Pulverisette 7 and Pulverisette 6 in the case of MA and RMA, respectively. To obtain comparable results for both milling processes, all the synthesis parameters were kept the same. The total mass of the pure starting powder mixture was 3.2 g. The masses of each starting material were calculated based on the studies of the starting materials to allow the formation of equimolar ScYNdGd alloy - in its metallic state (the actual ratio of mixed Sc-H, Y-H, Nd, Gd-H starting materials was 1.05:1.02:1.00:1.02). The milling processes were run at 600 rotations per minute (RPM). The ball-to-powder ratio (BPR) was 20:1. In both cases, 16 hardened steel balls of 10 mm diameter were used. The only slight difference between MA and RMA processes was the milling vial volume. 46 mL stainless steel vial was used for MA, while a 50 mL vial prepared from the same material was employed in RMA.

The MA process, which was performed in the Ar atmosphere, was stopped every hour to dissipate generated heat, hand-scrape the material from the milling media as well as collect the small sample for the XRD experiment (20 mg). The MA lasted in total 5 h.

The RMA process was run under 30 bar H_2. The process, which lasted for a total of 1 h, was interrupted every 15 minutes to collect the sample for XRD and differential scanning calorimetry/thermogravimetry (DSC/TG) experiments (50-100 mg). After each break, the vial was refilled with fresh hydrogen. Both samples, after the synthesis, were stored in the glovebox.

Material characterization. XRD was used to follow the evolution of the structure at different stages of MA and RMA and to evaluate the effect of heat treatment processes as well as hydrogenation and dehydrogenation on the structure of the alloy. The diffraction patterns were collected on an STOE STADI P transmission diffractometer using Mo radiation (0.7093 Å). The instrument was equipped with a primary Ge (111) monochromator ($MoK\alpha_1$) and a position-sensitive Mythen1K detector. The data were collected with a step width of 0.015° 2θ. Measuring times per step was 20 s. For each

measurement, eight scans were collected and summed after data collection. For the measurements, the samples were filled into borosilicate glass capillaries (0.3 mm) inside a glove box. The selected measured patterns were compared with simulated data using the crystal structure data taken from the ICSD database. Pawley fitting and Rietveld refinement was performed using TOPAS Version 6 (Bruker AXS, Karlsruhe, Germany). For Pawley fitting, simple cubic structures (Fm-3m and Im-3m) were assumed. The instrumental broadening was determined by an external silicon standard (NIST 640e), considering the simple axial model and the Thompson-Cox-Hastings pseudo-Voigt peak shape function.

The hydrogenated sample was used for in situ XRD measurement on the STOE STADI P in an STOE high-temperature attachment for capillaries. The hydrogenated MEA sample was mixed with a quartz powder in a weight ratio of 40:60 (to reduce absorption), then mixed with 20 wt% Si NISTe internal standard and filled into a 0.5 mm quartz capillary. The open capillary was kept under a constant flow of Argon to prevent air from coming in contact with the sample. A 5 °C/min heating rate was used with isothermal parts of 21 min at every 50 °C until 900°C. Three scans were collected at each step between 11 and 28.8° 2θ with a step width of 0.015° 2θ. The measuring time per step was five seconds. If the three scans were comparable, they have been summed up.

The morphology of alloy particles in as-synthesized and hydrogenated states was examined by scanning electron microscopy (SEM) – TM3030 (with an accelerating voltage of 15 kV) and Hitachi S-5500 (with an accelerating voltage of 30 kV). The chemical composition of the MA sample and distribution of elements were evaluated using Energy Dispersive X-Ray Analysis (EDX) – Xplore Compact 30 (Oxford) and Thermo Scientific UltraDry (SSD). The samples were placed on a copper grid with a lacey (carbon) film or an aluminum stub with a Leit-Tab (carbon and sulfur). The detailed microstructural observations of the as-synthesized and hydrogenated samples were done using Talos F200X STEM (Thermo Scientific) operating with an acceleration voltage of 200 kV. The microscope was equipped with a high-brightness field emission gun. In these experiments, the samples were placed on Cu lacey carbon grids. The images were obtained using transmission electron mode on thin particles or edges of the particles (parts thin enough to perform good quality analysis of transmitted electrons). Milling of particles with a focused ion beam was not applied.

The thermal analysis of the as-synthesized and hydrogenated samples was performed using Mettler Toledo TGA/DSC 1 STARe System thermogravimetric differential scanning calorimetry. All experiments were conducted on ~ 15 mg of material placed inside a 70 µL AlO_x sample crucible and run under 50 mL/min Ar gas flow. Samples were measured with a heating rate of 5 °C/min. In selected cases, the gas outlet of the DSC/TG has been connected to the ThermoStar GSD 300 T2 mass spectrometer (MS) to record the gas desorption profiles during the heating process. Some of the samples measured by DSC/TG were transferred in argon to the glovebox to be further studied by XRD.

The hydrogenation process at different H_2 pressures was studied by a high-pressure differential scanning calorimeter (HPDSC) - Mettler Toledo HP DSC 1. All measurements were performed on ~ 30 mg of material using a 40 µL aluminum sample crucible with a heating rate of 10 °C/min. The device was also used to perform cyclic

hydrogenation/dehydrogenation experiments. In this case, the hydrogenation was always performed under 15 bar H_2, while the dehydrogenation was run under 20 mL/min Ar gas flow. More details about experiments using HPDSC can be found in (S1).

The volumetric hydrogenation studies were performed using SETARAM PCT Pro Sieverts' apparatus. The mass of the studied sample was 1 g. The sample was placed inside a stainless-steel autoclave and then evacuated for 10 minutes under a dynamic vacuum at room temperature. No degassing/activation procedure was applied prior to the hydrogenation experiments. The sample holder and reservoir volume were 12.12 mL and 17.23 mL, respectively. In the first place, kinetic hydrogenation experiments were performed under different temperatures. The sample was degassed under a dynamic vacuum at 400 °C between the hydrogenation experiments. The cycled sample was used to obtain the PCI curve at 200 °C. After the experiment, the sample in a hydrogenated state was transferred to the glovebox and used in further experiments.

Thermal desorption spectroscopy (TDS) measurements have been performed on the hydrogenated sample under high vacuum conditions utilizing a quadrupolar mass spectrometer. More detail on the TDS setup and calibration can be found in (S2). The desorption experiment has been performed with a linear heating rate of 0.6 °C/min from RT up to 750°C.

The change of the mass due to the oxidation during the exposure of the samples to the air was studied with a Mettler-Toledo microbalance with a readability of 0.1 µg. Around 20 mg of each studied material has been closed hermetically in an Al crucible inside the glove box to avoid oxidation during handling. Each filled crucible was separately placed on the microbalance to weigh the accurate mass of a sample. Afterward, a small hole was made in the top part of the crucible to allow the sample to come into contact with the air, and the mass of the sample was measured over time.

DFT calculations. First-principles calculations based on density functional theory (DFT) are performed with the generalized gradient approximation proposed by Perdew, Burke, and Ernzerhof (GGA-PBE) as implemented in the VASP package.[S3,S4] We use the Projected Augmented-Wave (PAW) method considering the following electronic states as valence: H's $1s^2$, Sc's $3p^6 4s^2 3d^1$, Y's $4s^2 4p^6 5s^2 4d^1$, Nd's $6s^2 5d^2$, and Gd's $6s^2 5d^1$ (with frozen f-electrons in the core for the last two).[S5] The energy cut-off is truncated at 500 eV and we employ a Γ-centered k-point grid of 4 × 4 × 4 for a supercell containing 32 metal atoms (i.e., eight formula units). Each data point is obtained by performing a full geometry optimization using a conjugated gradient algorithm until the forces on the ions are smaller than 0.01 eV/Å. Hydrogenation of the system is considered by allocating hydrogen atoms (eight hydrogen atoms at each interval of H/M = 0.25) in random tetrahedral (and/or octahedral) positions.

For any composition $MH_x(x\epsilon[0,3])$, the calculation of the formation enthalpies ΔH_f and average binding energies $E_{B,avg}$ is done using the expressions:

$$\Delta H_f(MH_x) = E(MH_x) - \sum_{i=Sc,Y,Nd,Gd} E(M_i) - x\frac{1}{2}E(H_2)$$

and,

$$E_{B,avg}(MH_x) = E(M) + x\frac{1}{2}E(H_2) - E(MH_x)$$

Where $E(MH_x)$ is the computed energy of the alloy (hydride), $E(M_i)$ is the computed energy of each individual metal in its pure state, and $E(H_2)$ the computed energy of the hydrogen molecule.

Additionally, we also present the sequential binding energy ($E_{B,seq}$) that takes into account the energy required to keep adding hydrogen to an already hydrogenated system:

$$E_{B,seq}(MH_x) = E(MH_{x-1}) + i\frac{1}{2}E(H_2) - E(MH_x)$$

where $E(MH_{x-1})$is the computed energy of the previous data point and i represents the fraction of hydrogen added.

2. Starting materials

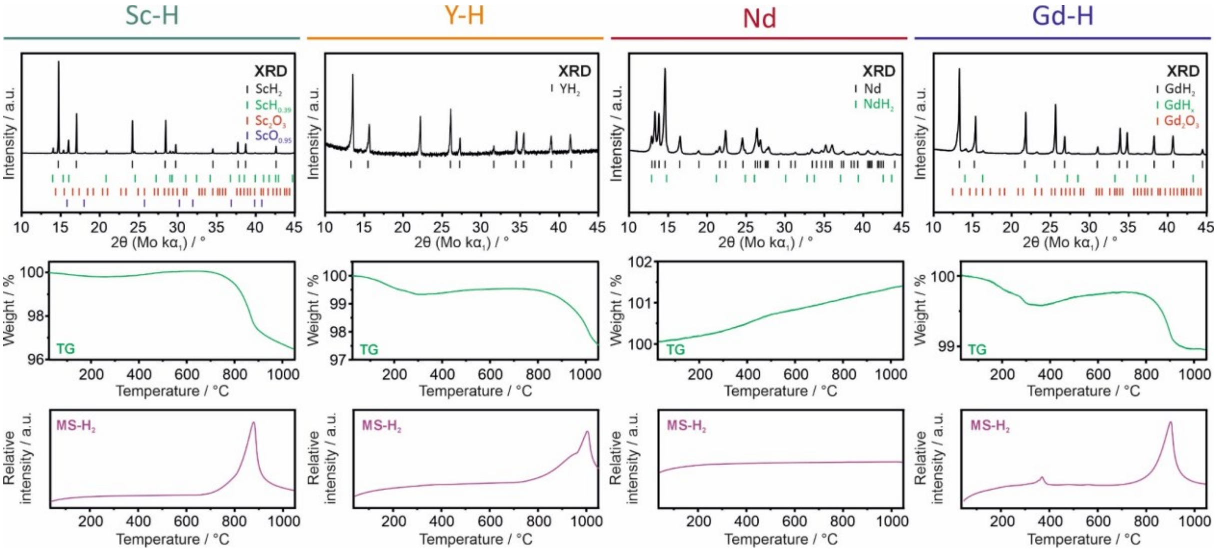

Figure S1 XRD patterns, TG, and MS (H_2) curves of the starting materials used in the synthesis of ScYNdGd alloy.

Figure S1 shows the XRD patterns and the TG/MS data (obtained at the heating rate of 5°C/min) of starting powders. All this data points out that the materials used in the synthesis were Sc, Y, and Gd dihydrides. Nd was used in its metallic state. The XRD analysis showed the presence of minor oxide or hydride phases in three of the starting materials.

Table S1 Refined weight fractions of each crystalline phase of starting materials (derived from Rietveld refinements of the XRD data).

Starting material	Phase	Space group	Lattice parameter / Å		Weight fraction / wt.%
			a	c	
Sc-H	ScH_2	Fm-3m	4.78348(3)		80(1)
	$ScH_{0.39}$	P63/mmc	3.3465(1)	5.3007(3)	16(1)
	Sc_2O_3	Ia-3	9.847(1)		2(1)
	$ScO_{0.95}$	Fm-3m	4.4948(7)		2(1)
Y-H	YH_2	Fm-3m	5.21228(9)		100.0(1)
Nd	Nd	P63/mmc	3.6582(1)	11.8001(5)	97(1)
	NdH_2	Fm-3m	5.4696(4)		3(1)
Gd-H	GdH_2	Fm-3m	5.30411(4)		89(1)
	GdH_x	Fm-3m	4.9806(7)		7(1)
	Gd_2O_3	Ia-3	10.824(2)		4(1)

3. Semi-empirical parameters for the design of MEAs

Over the years of research on HEAs and MEAs, several semi-empirical parameters (ΔH_{mix}, δ_r, Ω, VEC) were developed to help estimate the designed alloy's crystal structure. In most cases when -15 kJ/mol $< \Delta H_{mix} <$ 5 kJ/mol, $\delta_r < 6.6\%$, and $\Omega > 1.1$, the alloy crystallizes in a single-phase solid solution structure.[S6-S7] Furthermore, when the mentioned conditions are fulfilled, the VEC might help predict the structure of the solid solution phase: BCC, FCC, and HCP. The formation of BCC and FCC solid solutions has been observed mainly for VEC ≤ 6.87 and VEC ≥ 8, respectively. The range of VEC between 6.87 and 8 is majorly covered by alloys characterized by at least two-phase structures as a mixture of BCC and FCC phases.[S8] The determination of the VEC range at which the HCP structure can be expected is challenging due to the scarce data for this group of MEAs and HEAs. In several cases of REE-HEAs, the HCP structure has been observed for alloys with VEC near 3.[S6-S7] The situation is quite different for TM-HEAs crystallizing in HCP structure, for which the VEC was in the range between 5.8 and 8.2,[S9-S12] overlapping at the same time with the region of the alloys crystallizing in BCC, FCC, and BCC/FCC structures.

Accordingly, to the presented discussion of empirical parameters, the proposed in this study ScYNdGd alloy (in its metallic form) is highly likely to crystallize as a single-phase solid solution alloy (ΔH_{mix} = 1 kJ/mol, δ_r = 4,65%, Ω = 18.7). Moreover, since it is entirely composed of REEs (which crystalize in hexagonal structure at room temperature) and its VEC equals three, it can be expected to crystallize in the HCP structure.

4. Mechanochemical synthesis and thermal stability

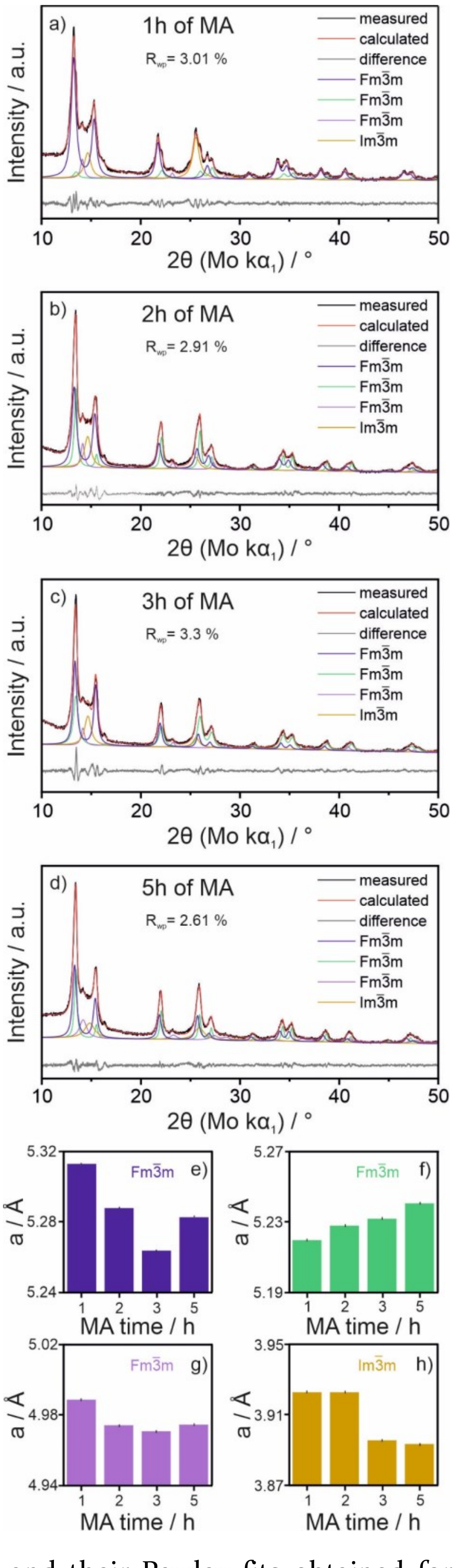

Figure S2 (a-d) XRD patterns and their Pawley fits obtained for the material mechanically alloyed for 1, 2, 3, or 5 hours, (e-h) lattice parameter of the phases present in the material as a function of mechanical alloying time. The colors of the bars in (e-h) correspond to the colors of the fitted structures.

Figure S2 a-d shows the XRD patterns obtained for the samples of material collected at different stages of the synthesis. The Pawley fits of the diffractograms suggest the formation of at least four cubic phases at each total milling time: three FCC phases and one BCC phase. It should be stressed that due to the complexity of Pawley analysis and the strong overlapping of the cubic phase patterns, the samples could be composed of more than four phases. Moreover, the very broad character of the reflections suggests not only the formation of small crystallites but also the possible broad distribution of lattice parameters of different FCC phases. Figure S2e-h shows the average values of the lattice parameter obtained for all the fitted phases. Due to the constant complexity of the phase composition of the studies system and lack of clearly exposed trends of lattice parameter changes during prolonged milling time, it is unlikely that the system would tend to create the single-phase solid-solution structure after continued milling.

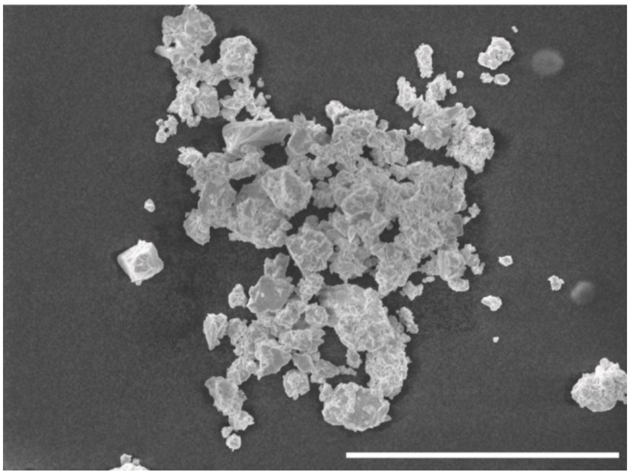

Figure S3 SEM micrograph (SE) obtained for ScYNdGd alloy after 5 h of MA (at 15 kV). Scale bar: 100 μm.

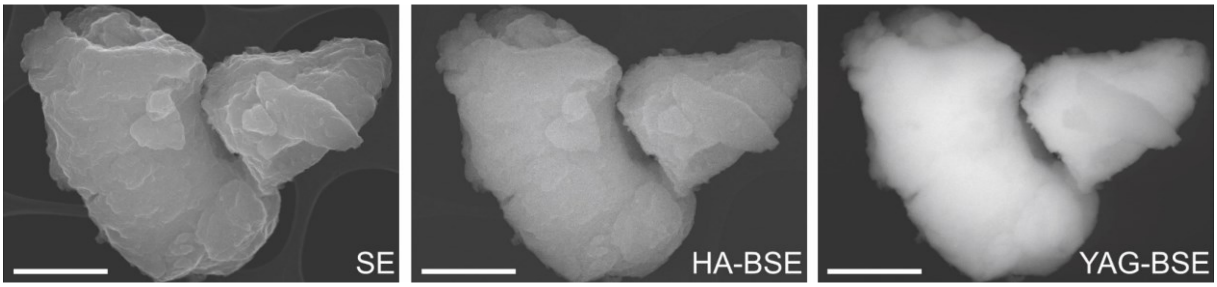

Figure S4 SEM micrographs obtained for ScYNdGd alloy after 5 h of MA (all at 30 kV). Scale bars: 1 μm.

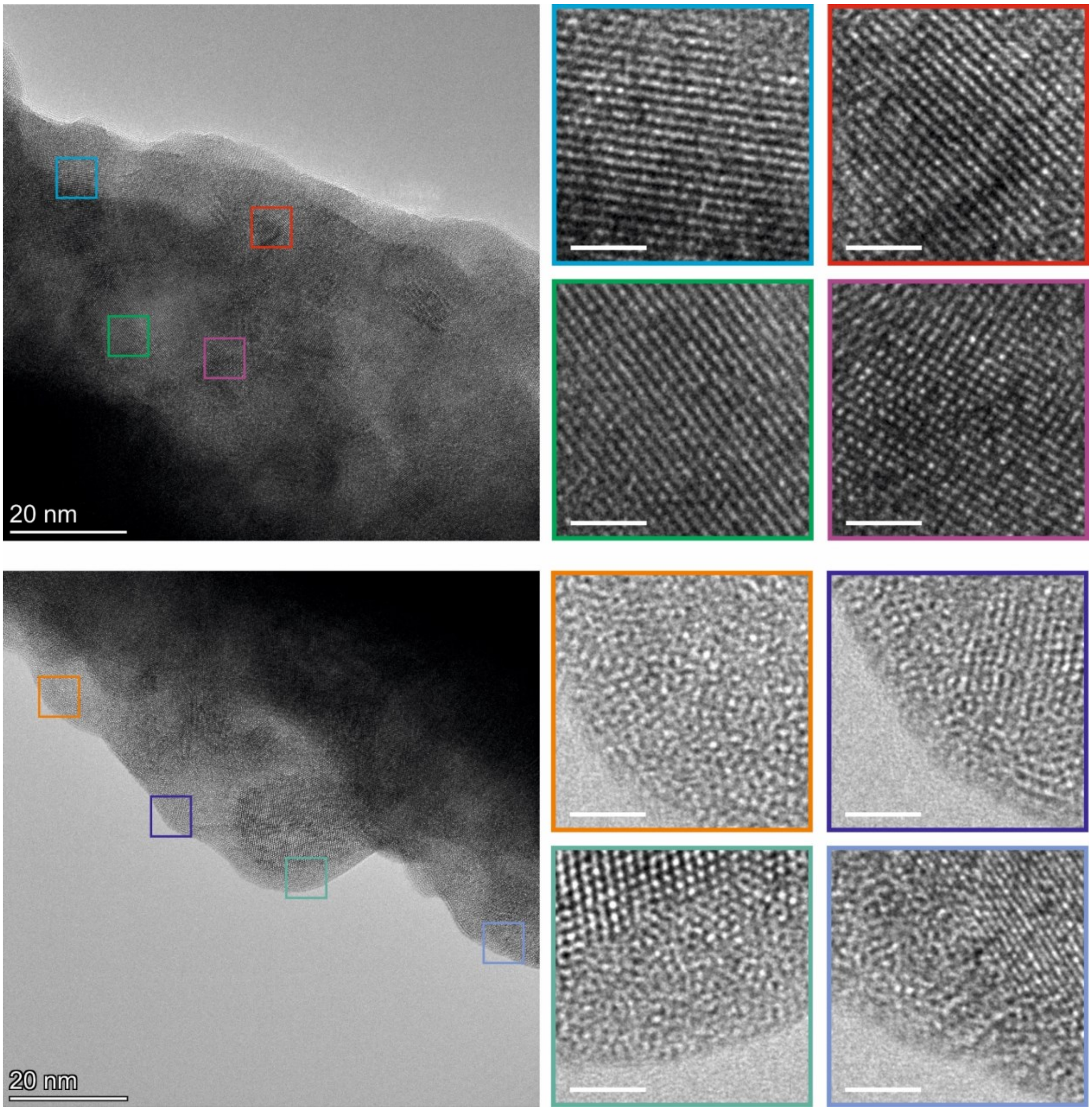

Figure S5 STEM micrographs obtained for ScYNdGd alloy after 5 h of MA (all at 200 kV). The upper part depicts the crystalline bulk part of the alloy, while the bottom part depicts the amorphous surface layer of the material. Scale bars in the magnified parts: 2 nm.

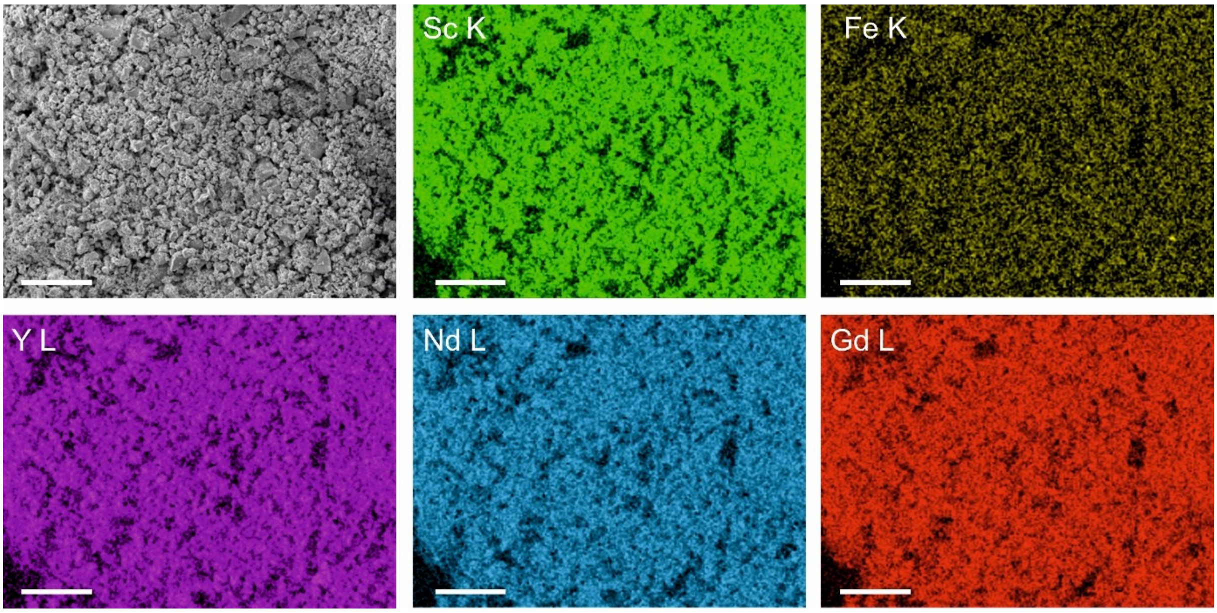

Figure S6 SEM micrograph (SE) and corresponding EDX images obtained for ScYNdGd alloy after 5 h of MA (all at 15 kV). Scale bars: 100 μm.

The designed and calculated (based on the EDX results) chemical compositions of the synthesized alloys are $Sc_{0.25}Y_{0.25}Nd_{0.25}Gd_{0.25}$ and $Sc_{0.23}Y_{0.22}Nd_{0.28}Gd_{0.27}$, respectively. The slight discrepancy between them could be related to the limitations of the EDX method – multiple scattering events present during investigations of particles or their agglomerates.

Moreover, the discrepancy could also originate from an inaccurate assumption regarding the chemical composition of the starting materials. The calculations of the amount of starting materials needed to obtain the equimolar ScYNdGd alloy were carried out based on the results of TG experiments of the starting materials - see Figure S1. TG curves provided information about the concentration of hydrogen stored in starting materials, which was implemented in the calculations. The inaccuracies in TG analysis automatically translate into inaccuracies in the mentioned calculations.

The discrepancy in the chemical composition should not be related to different inclinations of the starting materials toward accumulation on the milling media. Due to the brittle character of the used starting compounds, the milled powder had no significant tendency toward accumulation over the entire synthesis process.

Fe atoms in the as-synthesized alloy were detected in the EDX analysis, which is typical for mechanically alloyed materials and originates from steel abrasions (coming from the milling media). The content of Fe contamination is significantly lower than 0.5 at% (below the quantification limits of the method).

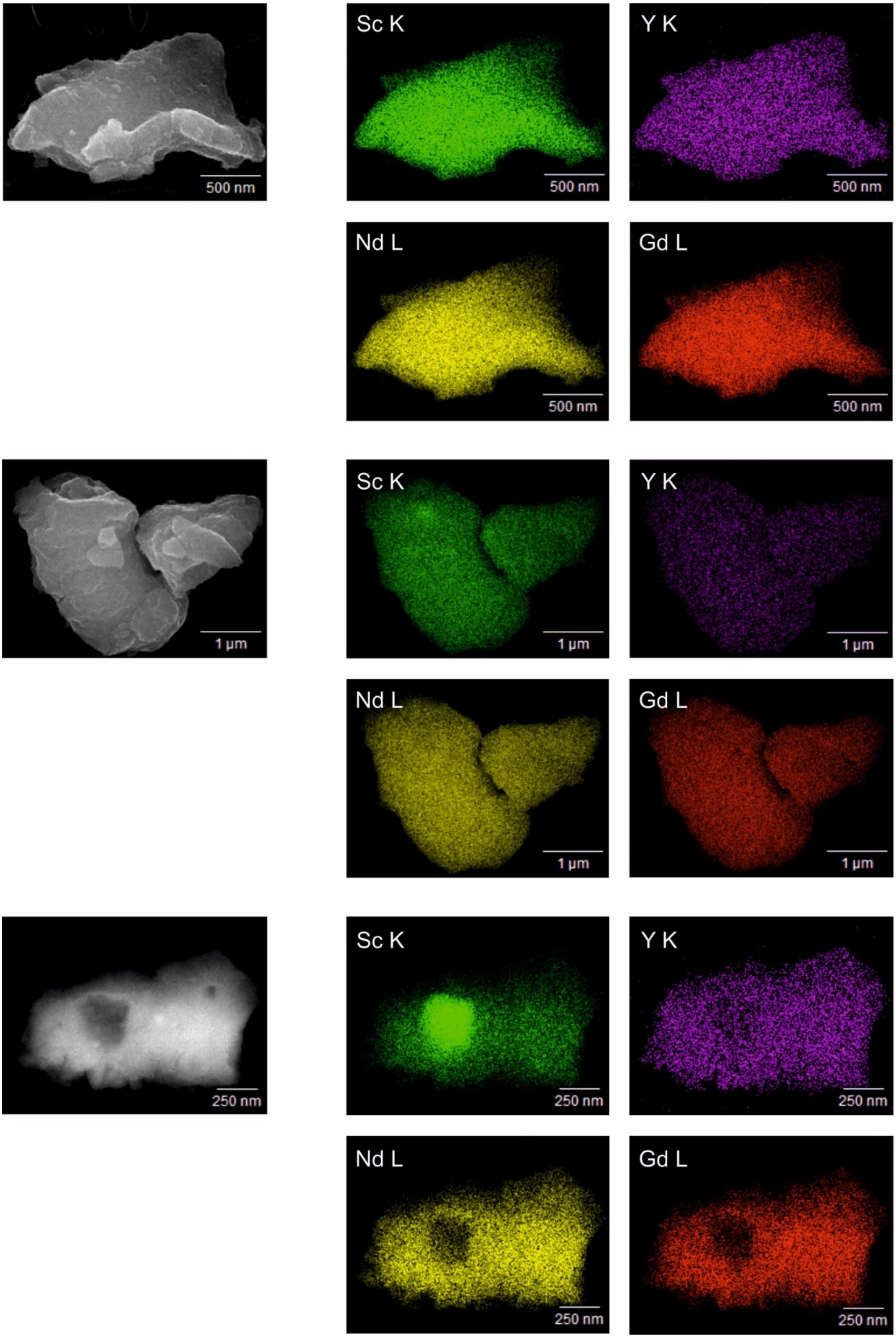

Figure S7 SEM micrographs (SE) and corresponding EDX images obtained for representative particles of ScYNdGd alloy after 5 h of MA (all at 30 kV).

5. First hydrogenation/dehydrogenation experiments

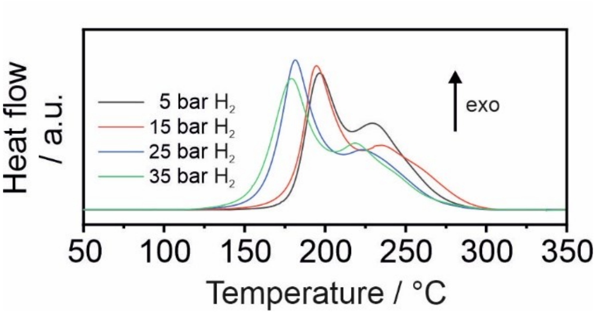

Figure S8 Results of hydrogenation HPDSC measurements at different H_2 pressures performed on as-synthesized alloy (after 5 h of MA).

The as-synthesized alloy was used for a series of HPDSC experiments performed at different hydrogen pressures during heating from 30 to 350 °C (Figure S8). The results showed that independent of the hydrogen pressure, the material absorbs hydrogen between 130 and 300 °C in a multistep reaction. The hydrogenation on-set temperature is decreased with the increase of the hydrogen pressure.

To check this tendency, we repeated the experiment seven times at 15 bar H_2 (Figure S9). In each case, the obtained curves show similar multistep shapes of the exothermic events but without repeatability regarding the on-set temperature. This shows that the hydrogenation on-set temperature does not depend on the hydrogen pressure (in the studied range of pressure) and is strongly determined by the measured sample (approximately 30 mg of as-synthesized alloy) - this results from the inhomogeneity of the chemical composition of particles and the presence of at least four phases in the as-synthesized alloy.

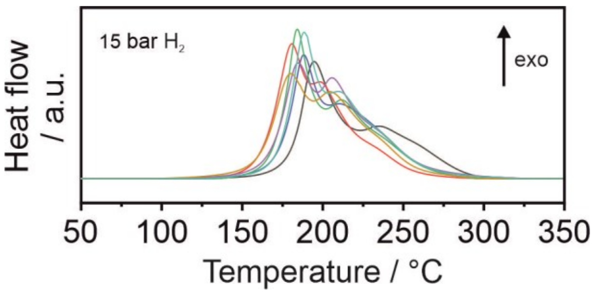

Figure S9 Results of hydrogenation HPDSC measurements at 15 bar H_2 performed on seven alloy samples after 5 h of MA.

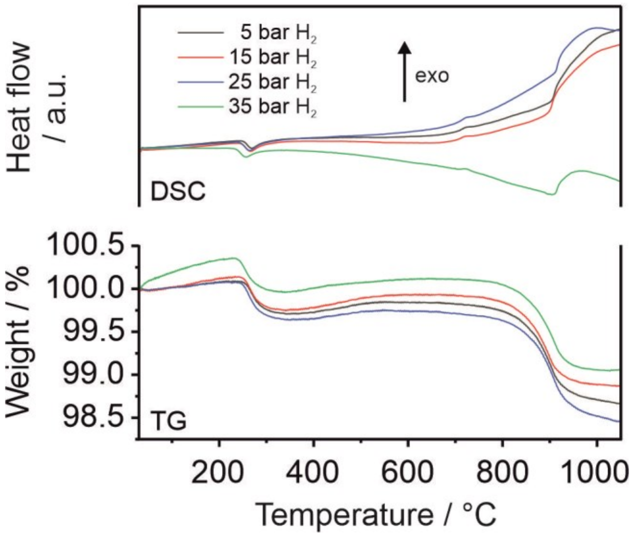

Figure S10 Results of dehydrogenation DSC/TG experiments performed on the samples hydrogenated using HPDSC (at different H_2 pressures).

The hydrogenated at different H_2 pressures (in HPDSC experiments – see Figure S8) samples have been further used in the dehydrogenation experiments employing TG/DSC/MS set-up (Figure S10). The curves showed a very similar decomposition process composed of a relatively low-temperature decomposition event at 220-400 °C and a high-temperature decomposition event at 700-1000 °C. The temperatures of the decomposition steps are comparable for all studied samples. TG curves show that the amount of hydrogen stored in the alloy after the hydrogenation does not depend on the hydrogenation pressure used – in the range of pressure between 5 and 35 bar H_2 (Figure S10, Table S2).

Table S2 Amount of hydrogen desorbed in the first and second dehydrogenation steps during DSC/TG experiments performed on samples hydrogenated in HPDSC measurements at 5, 15, 25, and 35 bar of H_2.

	**5 bar**	**15 bar**	**25 bar**	**35 bar**
**1st dehydrogenation step / wt.%**	0.37	0.39	0.43	0.40
**2nd dehydrogenation step / wt.%**	1.18	1.05	1.30	1.07

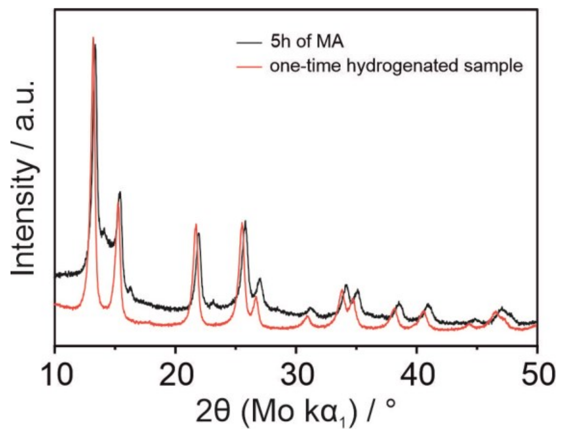

Figure S11 XRD patterns obtained for as-synthesized (after 5h of MA) and one-time hydrogenated ScYNdGd. To facilitate their comparison, both patterns were normalized according to the intensity of the most intensive reflection.

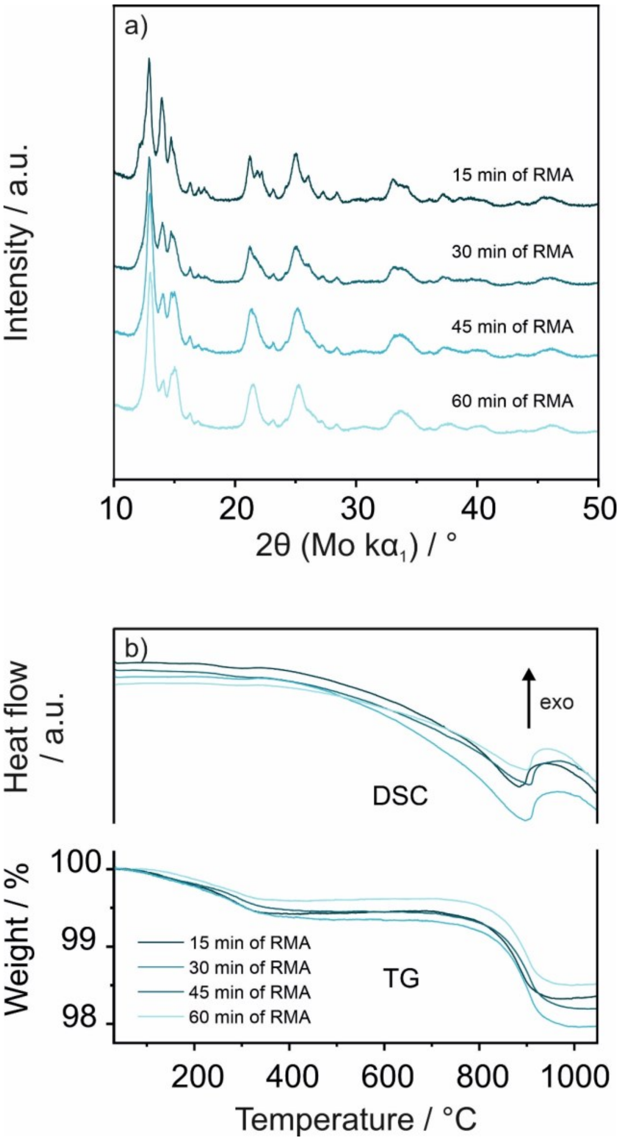

Figure S12 a) XRD patterns obtained for Sc-Y-Nd-Gd element mixture at different stages of the RMA process (milled at 30 bar H_2). b) DSC and TG results obtained for samples after different stages of RMA (5 °C/min).

6. Oxidation, activation and reactivation of alloy

The presented and discussed TG curves (Figures 1b, 2b, S10) exhibit regions at which the mass of the studied samples was raised. The phenomenon cannot be connected to the reactions taking place within the alloy and must be related to the apparatus and measurement conditions. Therefore, we looked at the possible reactions that could take place during the TG/DSC/MS experiments. The most obvious is the oxidation of the sample particles' surface. Particle oxidation is highly possible as the samples for TG/DSC/MS experiments were placed in open AlO_x crucibles, creating many opportunities for air contact during their handling. Moreover, even if highly purified Ar is used as the flowing gas during the experiment, the samples might be oxidized – especially those prone to it.

To check whether the surface of the studied alloy is easily oxidized, we designed a series of experiments to check the increase of the sample's mass over the time of exposure to the air (Figure S13, S14). Three different samples were studied: after 5h of BM, after 1 h of RMA, and hydrogenated sample (after a series of experiments with Sieverts' apparatus). The results show that all the samples are prone to oxidation. The gained weight depends on the sample and is extremely high for the RMA sample, reaching more than 2.5 wt.%. Such a tremendous increase in the mass must be connected with the synthesis process applied. First of all, the MA, irrespectively on the milling atmosphere, creates a lot of fresh surfaces that can be easily oxidized. Secondly, the hydrogen atmosphere used in RMA can be strongly reducing in the milling process, leading to a reduction of the surface oxides. Even if the mass increases observed for the BM and hydrogenated sample are comparably lower than those observed for RMA, both should be considered significant – particularly in comparison to the studied in this work weight loss related to the desorption of hydrogen.

The TG curves in Figure S14 show that the temperature regions at which the mass increases occur differ from sample to sample. This is because the increase in the mass is only detectable when the dehydrogenation process is not taking place simultaneously. Therefore, it can be concluded that samples can be oxidized throughout the entire TG/DSC/MS experiment – from 30 up to 1050 °C, especially at the beginning of the dehydrogenation process (Figure S14f). Conclusively, the measured gravimetric hydrogen capacities in this work may not be the actual ones but be affected/reduced by the contrary oxidation process.

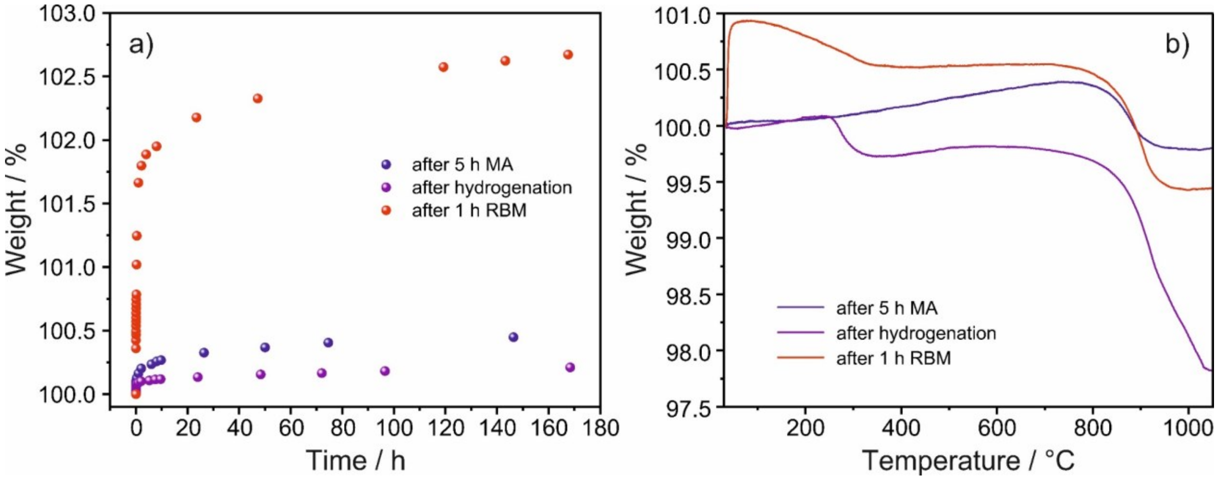

Figure S13 Change of the mass of the sample during exposure to the air (a) and TG curves during heating with 5 °C/min under Ar flow (b) of ScYNdGd alloy: after 5 h of MA, after hydrogenation (more specifically after a series of experiments using Sieverts' apparatus), after 1 h of RMA.

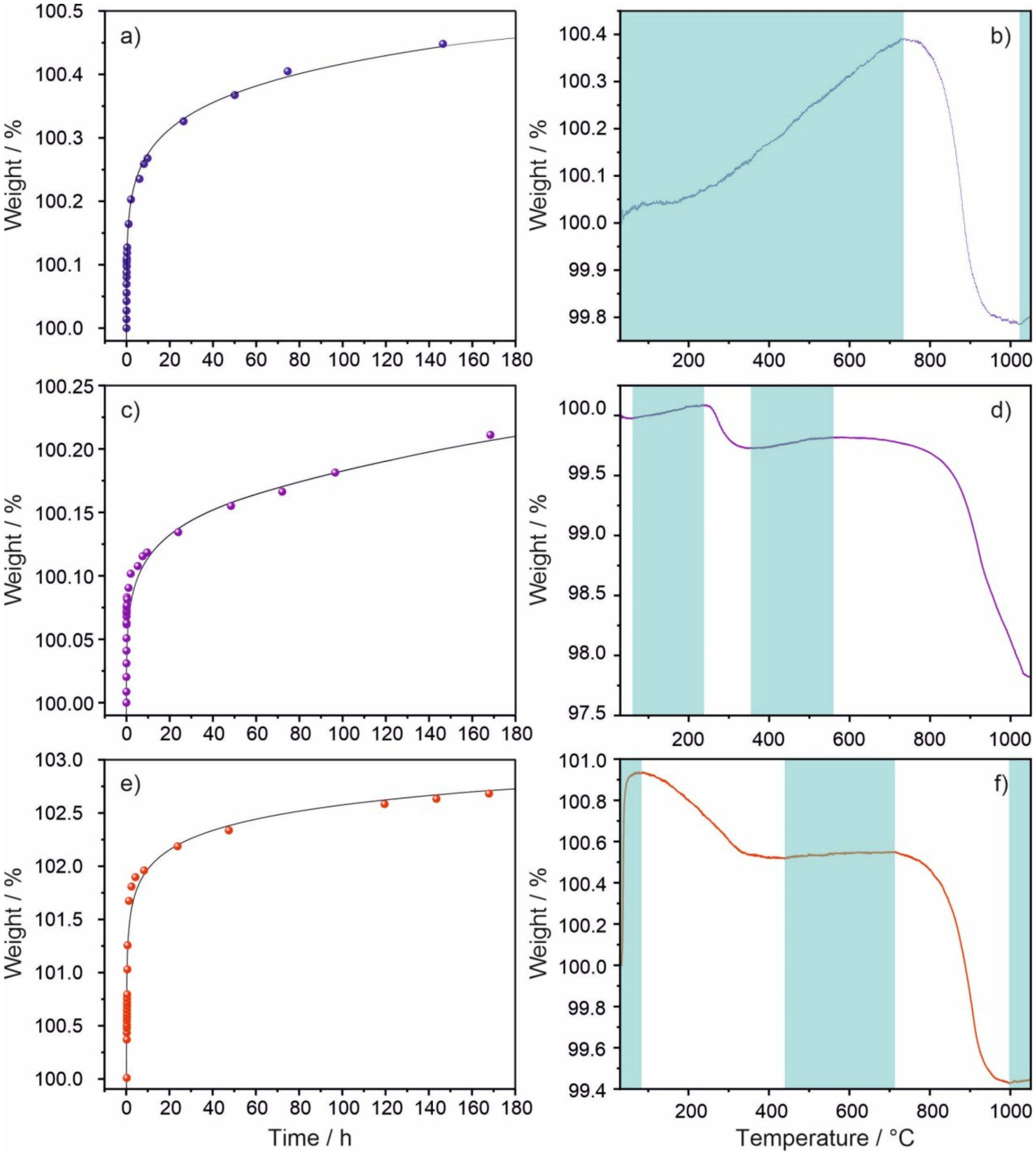

Figure S14 Change of the mass of the sample during exposure to the air (a,c,e) and TG curves during heating with 5 °C/min under Ar flow (b,c,f) of ScYNdGd alloy: after 5 h of MA (a,b), after a series of experiments using Sieverts' apparatus (c,d), after 1 h of RMA (e,f). Light blue areas correspond to the temperature ranges where the mass of the sample increased due to oxidation.

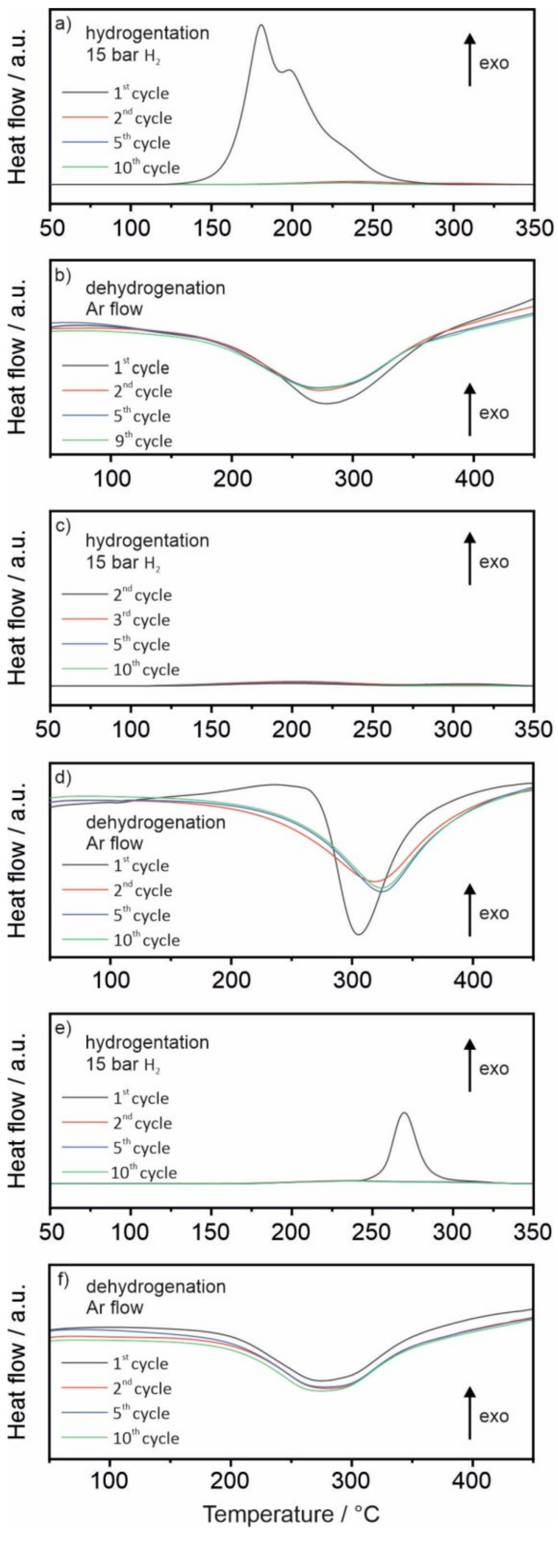

Figure S15 Representative hydrogenation HPDSC (a,c,e) and dehydrogenation DSC (b,d,f) curves obtained at different cycles of hydrogenation/dehydrogenation process for ScYNdGd alloy: a,b) after 5 h of MA, c,d) in the activated and hydrogenated state left for six-month long surface oxidation in air, e,f) in the activated and dehydrogenated state left for six-month long surface oxidation in air. All curves have been normalized according to the mass of the material used in the experiment.

The hydrogenation and dehydrogenation process in the following hydrogenation/dehydrogenation cycles performed on as-synthesized alloy has been studied by DSC (Figure S15a,b). The curve obtained during the first hydrogenation at 15 bar H_2 shows an exothermic peak which represents the heat generated during the hydrogen absorption process. The endothermic peaks visible in Figure S15b are related to the absorption of heat necessary to desorb hydrogen in the γ→β reaction.

Surprisingly, no exothermic peak was observed during the second hydrogenation process (Figure S15a). However, the endothermic dehydrogenation reaction could be observed in the second cycle (Figure S15b), consequently, the sample had to be firstly hydrogenated even if the process was not detected during the HPDSC measurement. The only explanation for this phenomenon could be: the first hydrogenation/dehydrogenation cycle led to the activation of the alloy. Thereby, the alloy could be fully hydrogenated in the second cycle already at the temperature of hydrogen insertion to the system (≤ 30 °C). This is before the HPDSC apparatus starts measuring because it needs several minutes to equilibrate at the starting temperature. This period is long enough to finish the hydrogenation process before the apparatus records any data.

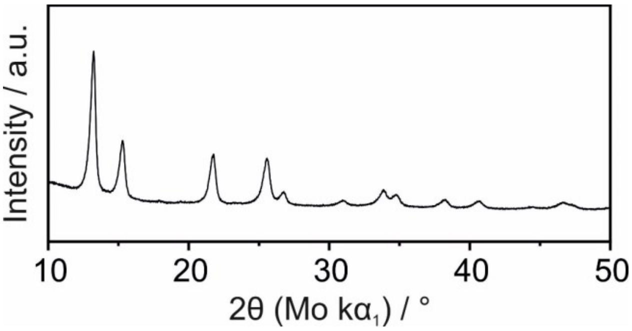

Figure S16 XRD pattern obtained for hydrogenated ScYNdGd alloy after ten hydrogenation/dehydrogenation cycles using HPDSC apparatus. Hydrogenation and dehydrogenation were performed under 15 bar H_2 and Ar flow, respectively.

7. Detailed hydrogenation/dehydrogenation studies

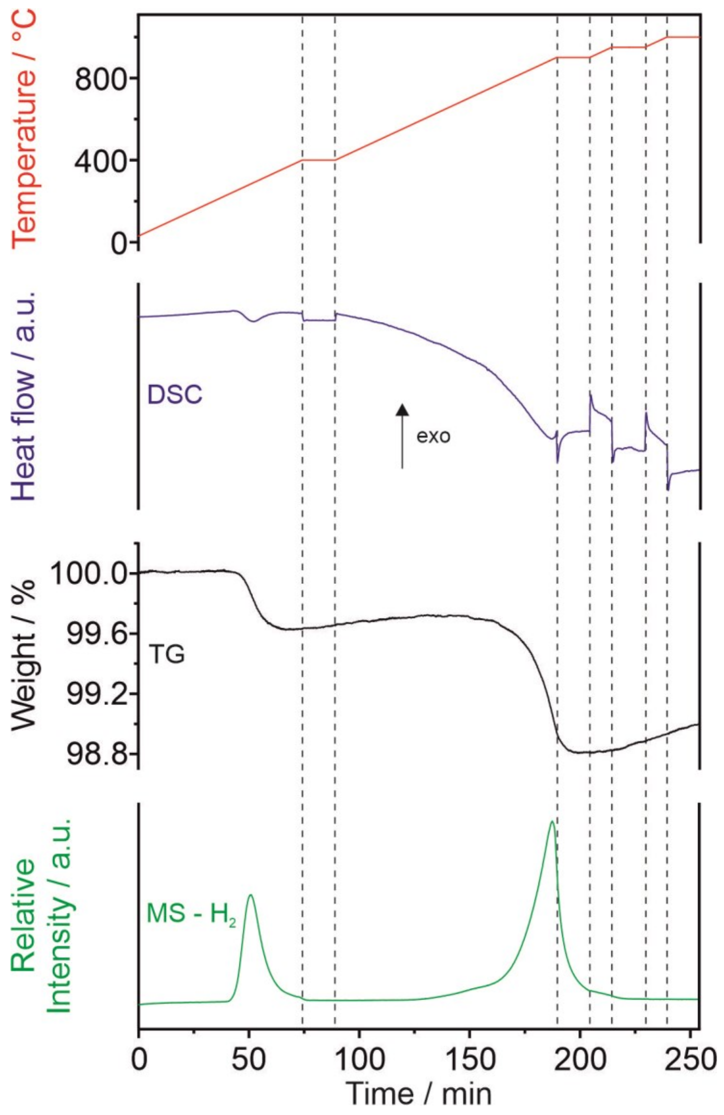

Figure S17 DSC, TG, and MS results obtained for hydrogenated ScYNdGd alloy during heating from 30-1000 °C (5 °C/min) with the 15-minute-long isothermal heating breaks at 400, 900, 950, and 1000 °C. The vertical dashed line indicates when isothermal heating periods started and ended.

Figure S17 shows the results of DSC, TG, and MS experiments performed on the hydrogenated ScYNdGd sample. The heating profile, which starts and finishes at 30 and 1050 °C, respectively, also contains the 15-minute-long isothermic parts. These time intervals at which the sample was kept at the constant temperature of 400, 900, 950, or 1000 °C were done to determine the lowest temperature necessary to finish each hydrogen desorption step. In the case of the first, low-temperature process, this information has a strong utilitarian character, answering the question of the possibility of finishing this desorption step using the Sievets apparatus (limited to 400 °C).

The presented data shows that the first step of the dehydrogenation process is nearly finished by reaching 400 °C. Moreover, the first minutes of isothermal heating at 400 °C ensures the completion of this decomposition step, and no further gas desorption is

observed in the continued heating above 400 °C. It proves that Sieverts' apparatus, whose operation temperatures are limited to 400 °C, can be used to volumetrically study the hydrogenation and dehydrogenation reactions occurring between di and trihydride of ScYNdGd alloy. Moreover, it should be emphasized that any desorption processes in Sieverts' apparatus can be supported by the dynamic vacuum, which in the vast majority of cases supports the desorption process, leading to faster hydrogen desorption and temperature reduction of this process.

The sample starts to desorb hydrogen within the second decomposition step at around 650 °C. Most of the hydrogen atoms are desorbed before 900 °C. The following isothermic heating at this temperature warrants the process's continuation but is insufficient to desorb all hydrogen within this step. Further heating to 950 °C with the following isothermal process is crucial to finish the complete dehydrogenation of the material.

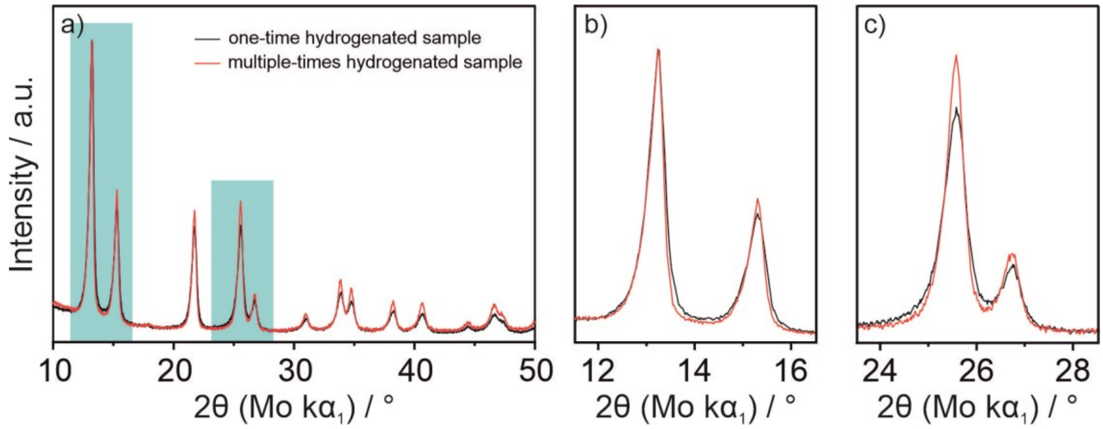

Figure S18 XRD patterns obtained for one-time and multiple-times hydrogenated ScYNdGd. The highlighted areas in part a) point out the 2θ range presented in more detail in parts b) and c). To facilitate their comparison, both patterns were normalized according to the intensity of the most intensive reflection.

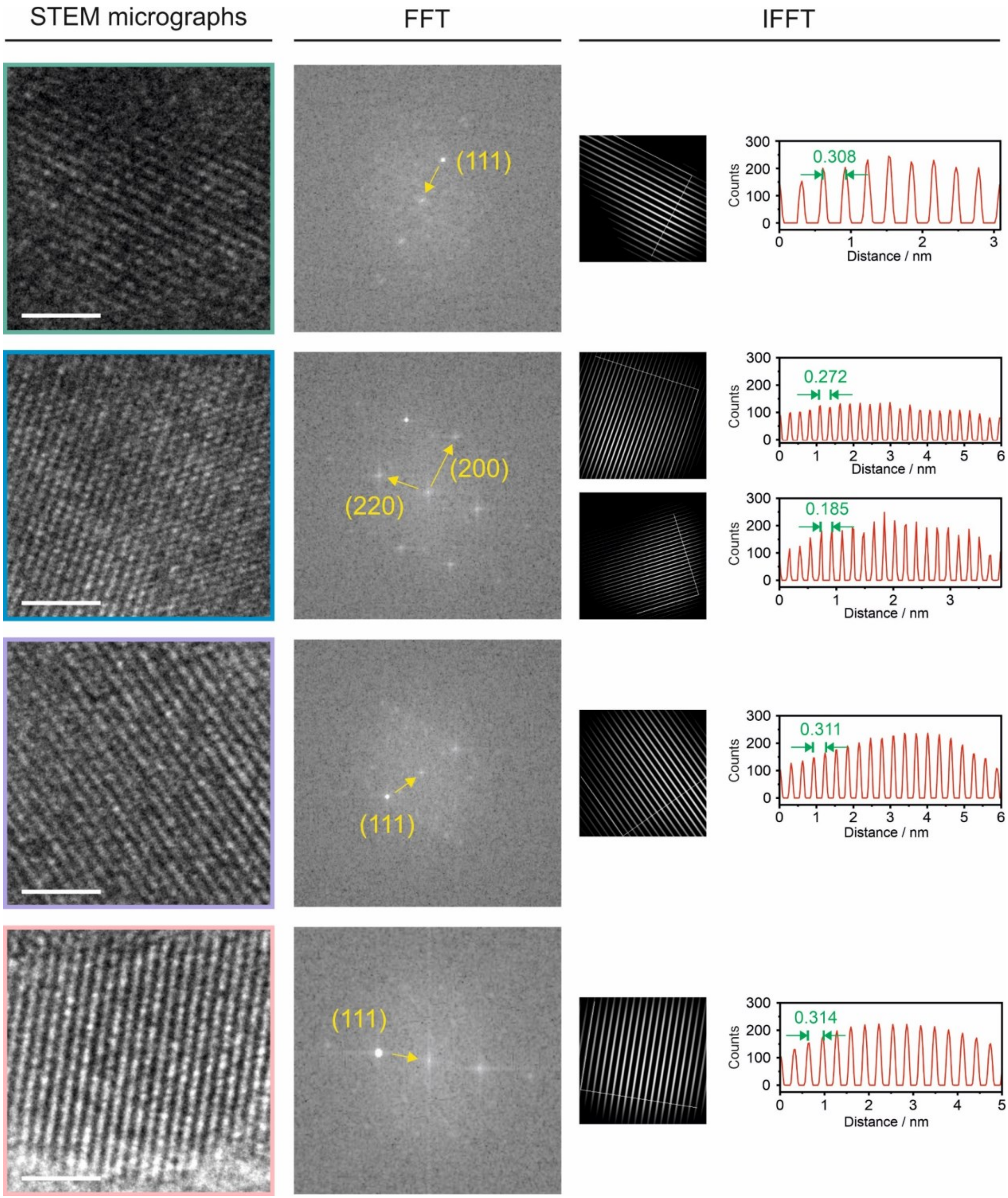

Figure S19 TEM micrograph, fast Fourier transform (FFT), and its lattice image transformed by inverse fast Fourier transform (IFFT) of hydrogenated ScYNdGd alloy. The particular planes and the d-spacing are indicated in Figure 5. Scale bars in the TEM micrographs: 2 nm.

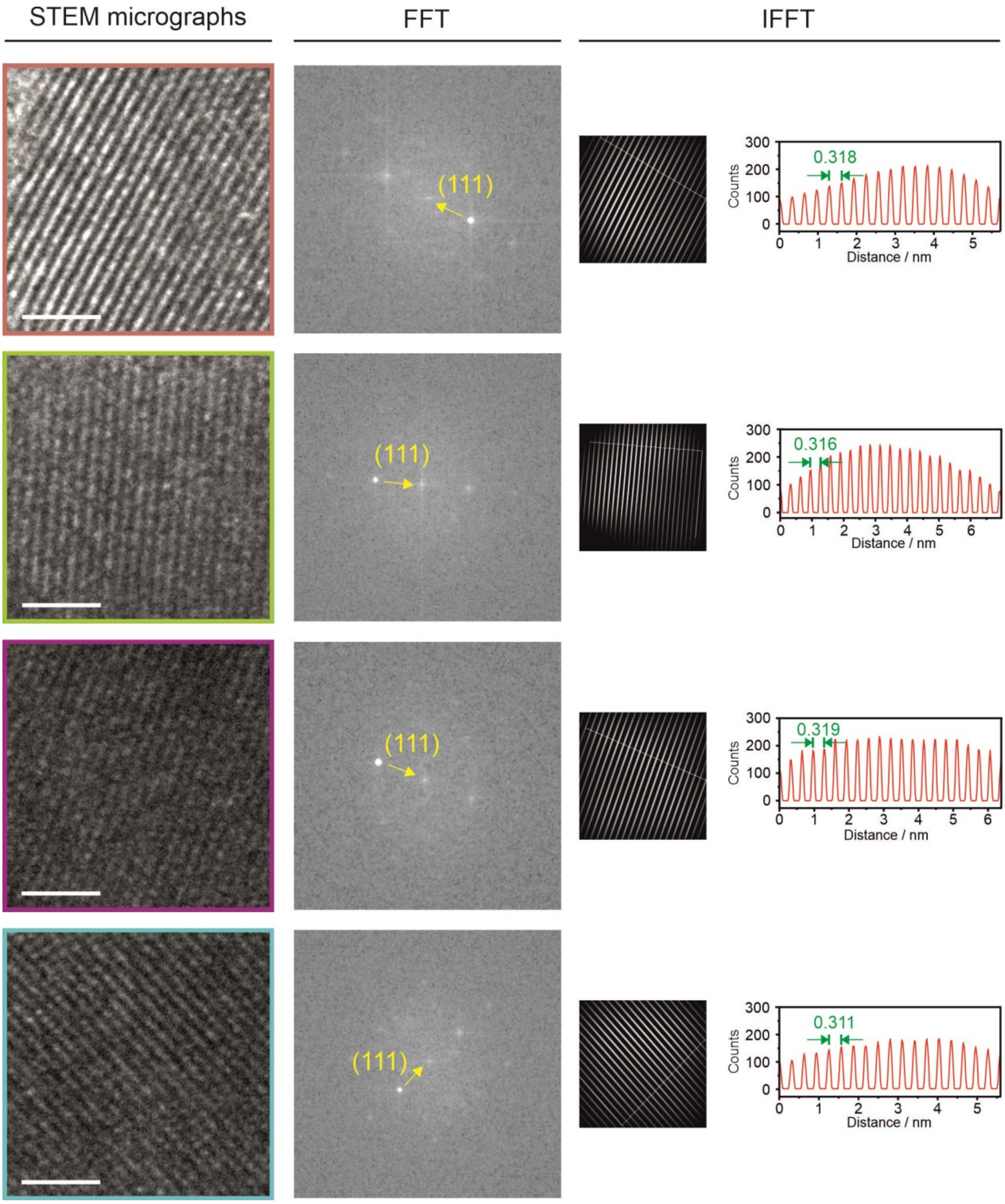

Figure S20 TEM micrograph, fast Fourier transform (FFT), and its lattice image transformed by inverse fast Fourier transform (IFFT) of hydrogenated ScYNdGd alloy. The particular planes and the d-spacing are indicated in Figure 5. Scale bars in the TEM micrographs: 2 nm.

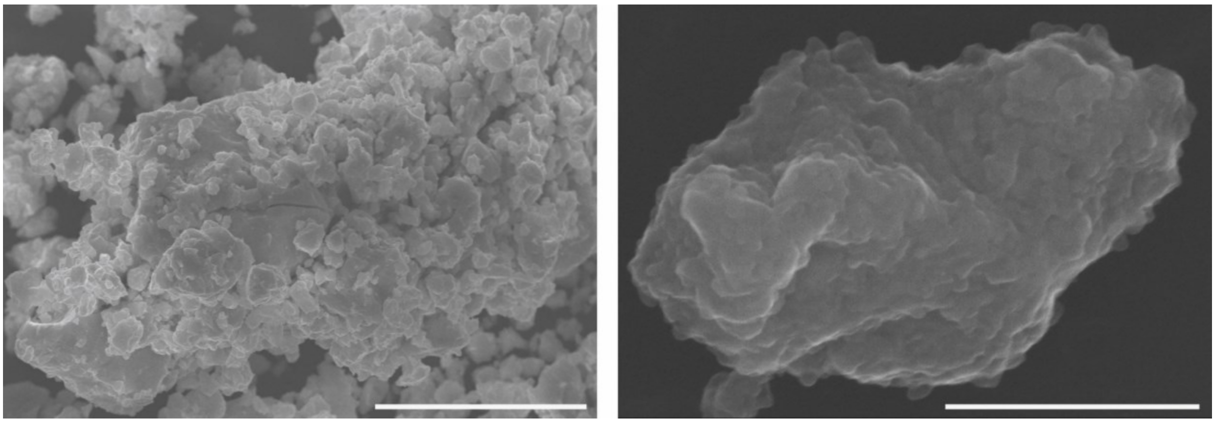

Figure S21 SEM micrographs obtained for hydrogenated ScYNdGd alloy after a series of experiments using Sieverts' apparatus (all at 30 kV). Scale bars: 30 μm (left), 300 nm (right).

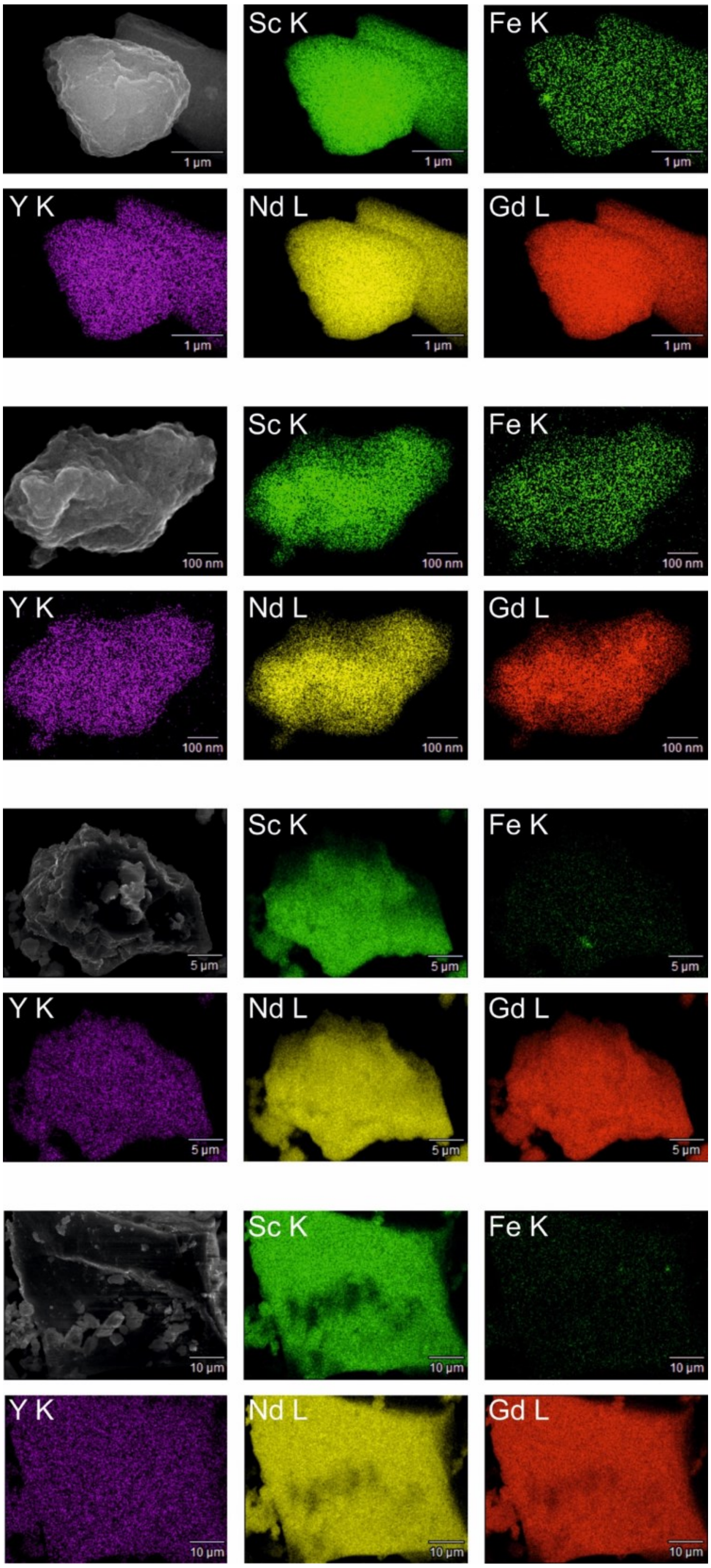

Figure S22 SEM micrographs (SE) and corresponding EDX images obtained for representative particles of hydrogenated ScYNdGd alloy after a series of experiments using Sieverts' apparatus (all at 30 kV).

References

(S1) Balcerzak, M.; Ternieden, J.; Felderhoff, M. Synthesis, thermal stability, and hydrogen storage properties of poorly crystalline TiVFeCuNb multi-principal element alloy, J. Alloys Compd. 2023, 943, 169142.

(S2) von Zeppelin, F.; Haluška, M.; Hirscher, M. Thermal desorption spectroscopy as a quantitative tool to determine the hydrogen content in solids, Thermochim. Acta 2003, 404, 251-258.

(S3) Perdew, J.P.; Burke K.; Ernzerhof M.; Generalized Gradient Approximation Made Simple, Phys. Rev. Lett. 1997, 77, 3865.

(S4) Kresse, G.; Furtmüller J. Efficient iterative schemes for ab initio total-energy calculations using a plane-wave basis set, Phys. Rev. B 1996, 54, 11169.

(S5) Blöchl, P.E. Projector augmented-wave method, Phys. Rev. B 1994, 50, 17953.

(S6) Steurer, W.; Single-phase high-entropy alloys – A critical update. Mater. Charact. 2020, 162, 110179.

(S7) Ye, Y.F.; Wang, Q.; Lu, J.; Liu, C.T.; Yang, Y. High-entropy alloy: challenges and prospects. Mater. Today 2016, 19, 349-362.

(S8) Vaidya, M.; Muralikrishna, G.M.; Murty, B.S. High-entropy alloys by mechanical alloying: A review. J. Mater. Res. 2019, 34, 664–686.

(S9) Marik, S.; Motla, K.; Varghese, M.; Sajilesh, K. P.; Singh, D.; Breard, Y.; Boullay, P. Singh, R.P. Superconductivity in a new hexagonal high-entropy alloy. Phys. Rev. Mater. 2019, 3, 602.

(S10) Yusenko, K.V.; Riva, S.; Carvalho, P.A.; Yusenko, M.V.; Arnaboldi, S.; Sukhikh, A.S.; Hanfland, M.; Gromilov, S.A. First hexagonal close packed high-entropy alloy with outstanding stability under extreme conditions and electrocatalytic activity for methanol oxidation. Scr. Mater. 2017, 138, 22-27.

(S11) Takeuchi, A.; Wada, T.; Kato, H. High-Entropy alloys with hexagonal close-packed structure in $Ir_{26}Mo_{20}Rh_{22.5}Ru_{20}W_{11.5}$ and $Ir_{25.5}Mo_{20}Rh_{20}Ru_{25}W_{9.5}$ alloys designed by sandwich strategy for the valence electron concentration of constituent elements in the periodic chart. Mater. Trans. 2019, 60, 2267-2276.

(S12) Lee, Y.-S.; Cava, R.J.; Superconductivity in high and medium entropy alloys based on MoReRu. Phys. C. 2019, 566, 1353520.
